# Searching and reporting in Campbell Collaboration systematic reviews: A systematic assessment of current methods

**DOI:** 10.1002/cl2.1432

**Published:** 2024-08-21

**Authors:** Sarah Young, Heather MacDonald, Diana Louden, Ursula M. Ellis, Zahra Premji, Morwenna Rogers, Alison Bethel, David Pickup

**Affiliations:** ^1^ University Libraries Carnegie Mellon University Pittsburgh Pennsylvania USA; ^2^ MacOdrum Library Carleton University Ottawa Canada; ^3^ University of Washington Health Sciences Library University of Washington Seattle Washington USA; ^4^ Woodward Library University of British Columbia Vancouver Canada; ^5^ Libraries University of Victoria Victoria Canada; ^6^ NHIR ARC South West Peninsula (PenARC) University of Exeter Medical School Exeter UK; ^7^ Centre for the Study of Learning and Performance Concordia University Montréal Canada

**Keywords:** Campbell Collaboration, evidence synthesis methods, information retrieval, MECCIR, reporting standards, systematic review

## Abstract

The search methods used in systematic reviews provide the foundation for establishing the body of literature from which conclusions are drawn and recommendations made. Searches should aim to be comprehensive and reporting of search methods should be transparent and reproducible. Campbell Collaboration systematic reviews strive to adhere to the best methodological guidance available for this type of searching. The current work aims to provide an assessment of the conduct and reporting of searches in Campbell Collaboration systematic reviews. Our objectives were to examine how searches are currently conducted in Campbell systematic reviews, how search strategies, search methods and search reporting adhere to the Methodological Expectations of Campbell Collaboration Intervention Reviews (MECCIR) and PRISMA standards, and identify emerging or novel methods used in searching in Campbell systematic reviews. We also investigated the role of information specialists in Campbell systematic reviews. We handsearched the *Campbell Systematic Reviews* journal tables of contents from January 2017 to March 2024. We included all systematic reviews published since 2017. We excluded other types of evidence synthesis (e.g., evidence and gap maps), updates to systematic reviews when search methods were not changed from the original pre‐2017 review, and systematic reviews that did not conduct their own original searches. We developed a data extraction form in part based on the conduct and reporting items in MECCIR and PRISMA. In addition, we extracted information about the general quality of searches based on the use of Boolean operators, keywords, database syntax and subject headings. Data extraction included information about reporting of sources searched, some aspects of search quality, the use and reporting of supplementary search methods, reporting of the search strategy, the involvement of information specialists, date of the most recent search, and citation of the Campbell search methods guidance. Items were rated as fully, partially or not conducted or reported. We cross‐walked our data extraction items to the 2019 MECCIR standards and 2020 PRISMA guidelines and provide descriptive analyses of the conduct and reporting of searches in Campbell systematic reviews, indicating level of adherence to standards where applicable. We included 111 Campbell systematic reviews across all coordinating groups published since 2017 up to the search date. Almost all (98%) included reviews searched at least two relevant databases and all reported the databases searched. All reviews searched grey literature and most (82%) provided a full list of grey literature sources. Detailed information about databases such as platform and date range coverage was lacking in 16% and 77% of the reviews, respectively. In terms of search strategies, most used Boolean operators, search syntax and phrase searching correctly, but subject headings in databases with controlled vocabulary were used in only about half of the reviews. Most reviews reported at least one full database search strategy (90%), with 63% providing full search strategies for all databases. Most reviews conducted some supplementary searching, most commonly searching the references of included studies, whereas handsearching of journals and forward citation searching were less commonly reported (51% and 62%, respectively). Twenty‐nine percent of reviews involved an information specialist co‐author and about 45% did not mention the involvement of any information specialist. When information specialists were co‐authors, there was a concomitant increase in adherence to many reporting and conduct standards and guidelines, including reporting website URLs, reporting methods for forward citation searching, using database syntax correctly and using subject headings. No longitudinal trends in adherence to conducting and reporting standards were found and the Campbell search methods guidance published in 2017 was cited in only twelve reviews. We also found a median time lag of 20 months between the most recent search and the publication date. In general, the included Campbell systematic reviews searched a wide range of bibliographic databases and grey literature, and conducted at least some supplementary searching such as searching references of included studies or contacting experts. Reporting of mandatory standards was variable with some frequently unreported (e.g., website URLs and database date ranges) and others well reported in most reviews. For example, database search strategies were reported in detail in most reviews. For grey literature, source names were well reported but search strategies were less so. The findings will be used to identify opportunities for advancing current practices in Campbell reviews through updated guidance, peer review processes and author training and support.

## 1 PLAIN LANGUAGE SUMMARY

Search strategies in Campbell reviews are generally good but grey literature searching and some standards need to be better reported.

### 1.1 The review in brief

Campbell Collaboration systematic reviews search a wide range of sources and grey literature and, in most cases, report detailed database searches and a complete list of sources. While search methods are generally good, there are some reporting standards that are often not met, possibly impacting the transparency and reproducibility of the review. Increased use of Campbell search guidance documents, the development of additional support for Campbell authors related to searching, and encouraging co‐author level involvement of information specialists may help improve conduct and reporting of searches in Campbell reviews.

### 1.2 What is this review about?

Campbell Collaboration systematic reviews aim to bring together knowledge from published and unpublished research to inform policy and decision‐making. Searching for studies is a key step in conducting a high quality review of the literature. Until now, no in‐depth assessment of the methods used for searching in Campbell Collaboration systematic reviews had been performed.

### 1.3 What is the aim of this review?

The current practices used by Campbell systematic review authors to conduct and describe their search methods for finding studies for their review were assessed. We wanted to know if Campbell review authors adhere to current standards and best practices for searching for systematic reviews and identify areas for improvement.

### 1.4 What studies are included?

We included Campbell systematic reviews published from 2017 until March 2024, which reported an original search strategy written in 2017 or later.

### 1.5 What are the main findings of this review?

In general, Campbell systematic reviews conduct broad searches in a wide range of databases and grey literature. Search strategies for databases were reported in most reviews, and show good practices related to the conduct of searches. There were some reporting standards that were less frequently met, such as reporting of grey literature searches, website URLs and database date ranges, impacting the reproducibility of searches. When information specialists are co‐authors, adherence to standards and quality improves for some search characteristics. There is also a time lag of about 20 months on average between the most recent search and the publication date.

### 1.6 What do the findings of this review mean?

While most Campbell systematic reviews conduct good quality searches across a wide range of databases and grey literature sources, there are some conduct and reporting standards that are not fully met by all reviews. These findings can help identify opportunities for improving current practices in Campbell reviews through updated guidance, peer review processes and author training and support.

### 1.7 How up‐to‐date is this review?

This review included all the Campbell systematic reviews published between January 2017 and March 2024.

## 2 BACKGROUND

### 2.1 Description of the problem or issue

In systematic reviews, searching for literature provides the foundational data set from which relevant studies included in the review will be drawn, and thus is a critical step in generating high‐quality, comprehensive and rigorous evidence synthesis products. Comprehensive, methodologically sound searches help to minimize bias and to ensure that all relevant evidence is found to inform the conclusions of the review. Clear and transparent reporting of search methods are essential for evaluating quality of a systematic review and for facilitating updates to reviews, which can become outdated not long after publication. While there is a substantial body of methodological conduct and reporting guidance for literature searching in systematic reviews, in practice, search conduct and reporting are often less than optimal, not transparent and/or not reproducible (Faggion et al., 2018; Koffel and Rethlefsen, 2016; Polanin, J.R. et al., 2020; Rethlefsen et al., 2024; Toews, 2017).

Existing systematic review guidance and standards provide conduct and reporting checklists that authors can follow to achieve consistent and reproducible searches. For Campbell systematic reviews, authors are often directed to the Methodological Expectations of Campbell Collaboration Intervention Reviews (MECCIR) Conduct and Reporting Standards (Methods Group of the Campbell Collaboration, 2019a, 2019b) and the Campbell Collaboration's guide to information retrieval (Kugley et al., 2017). The MECCIR standards were updated in late 2023 and were simplified, with conduct and reporting standards merged into a single document (Wilson et al., 2023). For the purposes of the current work, we used the previous MECCIR standards, which included mandated and highly recommended items related to the search for studies. For reporting, these standards indicated that authors must include a list of all sources searched along with the database name, platform and date coverage, an exact search strategy for each database searched, and justifications for search limits, among other requirements (Methods Group of the Campbell Collaboration, 2019b). The MECCIR conduct standard outlined required steps in the search for studies, including searching grey literature and searching reference lists of included studies, and highly recommended contacting experts for additional studies, among other conduct items (Methods Group of the Campbell Collaboration, 2019a). Additional standards that might be used by Campbell authors include Preferred Reporting Items for Systematic Reviews and Meta‐Analyses (PRISMA), updated in 2020 (Page et al., 2021). An extension to PRISMA, PRISMA‐S, was published specifically for search reporting (Rethlefsen et al., 2021), which provides detailed reporting items for transparent and reproducible searches. In addition, AMSTAR 2, a widely used critical appraisal tool for systematic reviews, includes ‘use a comprehensive literature search strategy’ as a checklist item required to meet the well‐established quality standards (Shea et al., 2017, p. 3).

The use of the above‐mentioned standards and guidelines for reporting in particular should help improve reproducibility and transparency in systematic review searches, which are important to demonstrate the comprehensiveness of the search and allow readers to assess the quality of the search, and thus, the results of the review (Rethlefsen et al., 2024). Reproducible and transparent methods highlight and reduce potential biases across the entire review process, from the search and selection of studies to data extraction, analysis and synthesis. Reproducible and transparent searches also highlight gaps that can be addressed in future work and facilitate the updating of systematic reviews, which is critical to incorporate new evidence as it emerges and to update recommendations and guidance that come from systematic reviews (Cumpston and Flemying, 2023). Understanding the degree to which Campbell systematic reviews follow recommended practices related to the reporting of searches can indicate how reproducible search methods are and shed light on areas for improving reproducible practices.

Finally, it has been shown that librarian co‐authors improve search reporting in systematic reviews (Aamodt et al., 2019; Meert et al., 2016; Pawliuk et al., 2024; Rethlefsen et al., 2015). Authors of Campbell systematic reviews can request editorial and methodological support and guidance to conduct searches. In some cases, information specialists or librarians with training in systematic searching are included as co‐authors or consultants. Campbell systematic reviews and protocols also undergo a peer review process that includes information specialists with expertise in systematic review searching who assess the appropriateness of the searched databases and grey literature sources, the usage of subject headings, keywords and Boolean operators, search syntax and the reporting of search methods, among other factors. No systematic assessment of information specialist and librarian involvement in Campbell systematic reviews has been conducted. The current study seeks to address this gap.

### 2.2 Description of the methods being investigated

We evaluated the current methods and reporting practices for source selection, searching, and reference management in Campbell Collaboration systematic reviews. We compared the conduct and reporting practices related to search and retrieval in these reviews to existing standards and guidelines including MECCIR (Methods Group of the Campbell Collaboration, 2019a, 2019b), PRISMA and PRISMA‐S (Page et al., 2021, Rethlefsen et al., 2021) and AMSTAR 2 (Shea et al., 2017). We also examined librarian or information specialist involvement in Campbell reviews and its relationship to aspects of the search as well as the appearance of any novel methods for the conduct of searching, such as text mining for keywords. Finally, it should be noted that the current assessment did not address search quality in terms of search performance, which is often assessed with measurements of precision and sensitivity. This aspect of searching is outside the scope of the current study.

### 2.3 Description of the condition

Not applicable.

### 2.4 Description of the intervention

Not applicable.

### 2.5 How the intervention might work

Not applicable.

### 2.6 Why it is important to do this review

No broad systematic assessment of the search methods used in Campbell reviews across all coordinating groups has been published to date and the extent to which Campbell review searches adhere to current guidelines is unclear. Ramirez et al., 2022 assessed adherence to MECCIR standards amongst Campbell reviews published by the Education Coordinating Group since 2014 and found that while some criteria were well followed by all reviews (e.g., searching grey literature, reporting database names and using keywords in search strategies), others proved less consistent with many reviews falling short of full adherence (e.g., reporting website URLs). Wang et al., 2021 conducted a review of methodological and reporting characteristics of Campbell systematic reviews across all coordinating groups published since 2011, but did not address detailed aspects of search conduct and reporting specifically. The current study aimed to build on this work focusing on search methods and reporting with an in‐depth examination of all search methodological and reporting aspects necessary to adhere to standards and to make searching reproducible, across all Campbell Collaboration coordinating groups.

## 3 OBJECTIVES

The aim of this study was to assess search methods and reporting across Campbell systematic reviews for all coordinating groups. Our specific objectives include examining how searches are currently conducted in Campbell systematic reviews, including identifying any new or emerging methods and approaches to searching, and to examine how search strategies, search methods and search reporting adhere to the 2019 Methodological Expectations of Campbell Collaboration Intervention Reviews (MECCIR) and 2020 PRISMA guidelines. We also aimed to examine the involvement of information specialists in Campbell reviews.

## 4 METHODS

### 4.1 Criteria for considering studies for this review

#### 4.1.1 Types of studies

Systematic reviews of all types and topics were included. Updates to previous systematic reviews were also included when changes to search methods were made. Protocols, methods papers, commentaries, editorials and other types of evidence synthesis (e.g., evidence and gap maps, mega maps) were excluded.

We chose January 2017 as a publication date limit to reflect our interest in examining current search practices. As search methods have evolved with rapid changes in the information landscape, reviews published since 2017 should provide an accurate picture of current practices. That being said, the date of publication of a review is not an accurate representation of when the search for studies was conducted. Reviews published in 2017 or later represent searches conducted in the years before and after 2017. These reviews provide a broader timeframe to look at the change in practices over time.

#### 4.1.2 Types of participants

Not applicable.

#### 4.1.3 Types of interventions

Not applicable.

#### 4.1.4 Types of outcome measures

Included reviews must have reported on an original search strategy developed for that review. For example, in the course of data extraction, we encountered a number of systematic reviews that relied on searches of the Global Policing Database (GPD) (https://gpd.uq.edu.au/s/gpd/page/about), as opposed to designing original search strategies for a range of sources. Since these reviews did not develop or report their own search strategies and instead pointed to the methods used to develop the GPD, we chose to exclude these reviews from this assessment. This was also true of reviews that relied on a search strategy originally developed for a separately published evidence and gap map, or update reviews that did not amend a search strategy for the review published in 2017 or later.

For included reviews, we recorded detailed information about the search‐related aspects of the review described in the sections below. For items related to adherence to standards and guidelines, we tried to capture levels of adherence (i.e., complete, partial and no adherence) to gain a better understanding of practices. Depending on the variable, we recorded either Yes/No or one of a set of answers reflecting a range of reporting and conduct. For example, whether authors searched at least two relevant databases was answered with a simple Yes/No response. On the other hand, whether authors reported the websites searched could be a range of answers including: all websites reported (i.e., full adherence to this reporting standard), some websites reported (i.e., partial adherence), none reported (no adherence), or not applicable (did not search websites). More details about how each conduct and reporting item was handled can be found in the data set in File S2.

##### Primary outcomes

We aimed to compare the conduct and reporting practices related to search and retrieval to existing standards and guidelines. Thus, primary measures of conduct and reporting included those required for adherence to MECCIR (Methods Group of the Campbell Collaboration, 2019a, 2019b), PRISMA and PRISMA‐S (Page et al., 2021; Rethlefsen et al., 2021) and AMSTAR 2 (Shea et al., 2017). This included both mandatory and highly desirable items from these standards and guidelines.

For source reporting and conduct, we recorded whether reviewers searched at least two relevant databases; reported complete lists of databases, grey literature sources and websites; provided database information including platform name and dates of coverage; and whether website URLs were reported. We also recorded whether handsearching and forward and backward citation searching were conducted, reference lists of related reviews searched and experts contacted.

For search conduct, we performed a general assessment about whether Boolean operators, phrase searching, subject headings, database syntax and keyword variations were used correctly, in alignment with MECCIR conduct expectations.

For search reporting, we recorded whether full search strategies were provided for all sources searched, including grey literature and supplementary methods as well as dates of searches and justifications for search limits. Primary measures also included whether the Kugley et al. (2017) Campbell searching guidance was cited.

##### Secondary outcomes

As we were also interested in understanding the role of information specialists in Campbell reviews, we assessed the presence of an information specialist as a co‐author, or acknowledgement of an information specialist in the methods, acknowledgements, or roles of authors section. We extracted additional details about reporting of methods for forward citation searching and contacting experts, as well as the use of reference management software. Finally, in the interest of understanding the use of any novel methods for search conduct, we recorded any mentions of methods used for term harvesting, running of searches, and deduplication beyond standard approaches (e.g., automation tools).

### 4.2 Search methods for identification of studies

We searched the *Campbell Systematic Reviews* journal via the Wiley Online Library website (https://onlinelibrary.wiley.com/journal/18911803) to identify all systematic reviews published since January 2017. The search was conducted initially in July 2021, updated in June 2022 and then again in March 2024. The search was conducted by handsearching the tables of contents of all *Campbell Systematic Reviews* issues from 1 January 2017 (volume 13 issue 1) and included issues up to 1 March 2024 (volume 20 issue 1).

#### 4.2.1 Electronic searches

Not applicable.

#### 4.2.2 Searching other resources

Not applicable.

### 4.3 Data collection and analysis

#### 4.3.1 Selection of studies

Given the straightforward nature of the selection criteria (i.e., systematic reviews or updates to systematic reviews published in January 2017 or later), a single reviewer conducted the search and selection of studies (S. Y.).

#### 4.3.2 Data extraction and management

A data extraction form was developed based in part on MECCIR reporting standards (Methods Group of the Campbell Collaboration, 2019b), PRISMA 2020 (Page et al., 2021) and the PRISMA‐S Extension for reporting literature searches in systematic reviews (Rethlefsen et al., 2021). An initial draft template was piloted by six authors on nine different systematic reviews. After this initial test, the form was modified to include a total of 79 items. The form was again piloted by two authors independently on five systematic reviews, and based on this and discussions with the author team, additional modifications and clarifications were made. Thus, the final data extraction form differs slightly from the form published with the protocol. The final version includes 90 extraction items and can be found in File S1 along with the associated variables names used in the data set.

Data about the following were extracted from each review:

1. Bibliographic characteristics of the reviews including: title, authors, year of publication, Campbell coordinating group associated with the review, whether the review was an update, and the most recent search date reported in the review.
2. Information sources that were searched including: whether bibliographic databases were listed, types of grey literature sources searched (conference proceedings, theses and dissertations, clinical trials and registries, and government and non‐government websites), and whether sources specific to geographic regions were searched.
3. Supplementary search methods including: citation searching, the use of search engines (e.g., Google), free scholarly search engines (Google Scholar, Microsoft Academic, Dimensions, or other), handsearching and contacting experts.
4. The reproducibility of searches including reporting of details about the sources searched (e.g., database platform, date coverage, etc.), search strategy reporting, reporting of grey literature searches, website URLs and methods for supplementary searches.
5. The quality of searches including appropriate use of Boolean operators, keyword variations, the use of subject headings, database search syntax, and use and justification of limits.
6. The use of reference management software and deduplication methods.
7. The involvement, or lack thereof, of an information specialist including: whether a librarian was a co‐author, acknowledged, or mentioned in the methods section.
8. Reference to the Campbell search methods guidance by Kugley et al., 2017.
9. The use of machine learning or automation tools for search and deduplication, as well as other emerging or less common methods.

Data was extracted from all included Campbell systematic reviews using a form developed in Google Sheets. Data extraction was carried out independently by two authors and discrepancies were resolved by a third author. For updated systematic reviews, if the update indicated changes to the search methods, then data was extracted from the updated systematic review for inclusion in subsequent analyses.

#### 4.3.3 Assessment of risk of bias in included studies

We used an adapted version of AMSTAR 2 (Shea et al., 2017), specifically item 4 related to the search, to assess risk of bias in Campbell Collaboration systematic reviews using this widely used critical appraisal tool. Item 4 in the AMSTAR 2 checklist asks ‘Did the review authors use a comprehensive literature search strategy?’. There are eight sub‐items required to achieve a Yes or Partial Yes rating for Item 4. Because AMSTAR 2 was developed for medical and health‐related systematic reviews, this includes the requirement to search trial/study registries. However, for reviews in the social sciences, such trial or study registries may not exist or be appropriate for searching. Thus, we adapted the AMSTAR 2 checklist by removing the ‘searching trial/study registries’ requirement for a Yes rating.

#### 4.3.4 Measures of treatment effect

Not applicable.

#### 4.3.5 Unit of analysis issues

Not applicable.

#### 4.3.6 Dealing with missing data

Not applicable.

#### 4.3.7 Assessment of heterogeneity

Not applicable.

#### 4.3.8 Assessment of reporting biases

Not applicable.

#### 4.3.9 Data synthesis

The extracted data was pre‐processed using a combination of OpenRefine and R/RStudio. Most of the analyses were carried out in R (version 4.2.1) and RStudio (version 2023.12.0 + 369). Descriptive statistics and summaries of the data were calculated for the variables related to conduct and reporting. This includes frequencies and percentages for categorical variables, and mean, median and range for numerical variables. For numerical variables (e.g., time lag in months between search date and publication date), we compared groups using one‐way Analysis of Variance (ANOVA).

Some categorical variables were extracted as binary Yes/No answers as to whether an item was conducted or reported. For other variables with a range of answers, answers were grouped into full, partial, or not conducted/reported (or not applicable) and these nuances were taken into consideration in the descriptive analysis and interpretation. For example, for whether authors reported dates of searches, they may have reported a general date range (e.g., ‘searches were run in May 2020’), exact search dates for each search, or no dates at all. In this case, general date range would be considered partial adherence to the standard to report the search dates for each source. To assess adherence to standards, study variables were aligned with the 2019 MECCIR standards and PRISMA 2020 standards. Depending on the variable, degree of compliance (full/partial/none) for each study variable was converted to a number (2/1/0) then assigned a colour (blue/yellow/red) to create a heatmap visualizing study variable adherence to the standards. This was carried out in Excel.

#### 4.3.10 Subgroup analysis and investigation of heterogeneity

We were interested in understanding differences across Campbell coordinating groups as these groups may have variable editorial and discipline‐specific processes and practices that could impact search reporting and conduct. We conducted sub‐group analyses by coordinating group for a number of variables, including types of grey literature searched, involvement of an information specialist, and time lag between most recent search date and publication date. For the time lag variable, we used one‐way Analysis of Variance (ANOVA) to determine whether there were statistical differences between coordinating groups.

We were also interested in whether the involvement of an information specialist as a co‐author versus through consulting or not at all was related to differences in search conduct and reporting. Thus, for reviews with an information specialist co‐author, that consulted an information specialist but without co‐authorship and those not mentioning information specialist involvement, we calculated the percentage adhering to different conduct and reporting best practices and standards.

#### 4.3.11 Sensitivity analysis

Not applicable.

#### 4.3.12 Summary of findings and assessment of the certainty of the evidence

Not applicable.

## 5 RESULTS

### 5.1 Description of studies

#### 5.1.1 Results of the search

One‐hundred and eleven systematic reviews or systematic review updates were published from January 2017 until the time of the most recent search (March 2024). Of these, 11 were updates to previous systematic reviews. A complete list of included studies, along with the extracted data and Study IDs, which are used for reference throughout the manuscript, can be found in File S2. All of the codes associated with the analyses can be found in Young, 2024.

#### 5.1.2 Included studies

The coordinating groups that published the highest number of systematic reviews were International Development, Education and Social Welfare, each with 29 reviews. The majority of reviews (83) did not relate to any specific geographic region. Twenty‐three were related to Low‐ and Middle‐Income Countries only. Three were concerned with the United States only.

Table 1 shows the characteristics of the included reviews.

**Table 1:** Characteristics of included studies.

Characteristics	Category	Number (%)
Coordinating group^a^	Ageing	1 (<1)
	Business and Management	1 (<1)
	Crime and Justice	24 (22)
	Disability	6 (5)
	Education	29 (26)
	International Development (incl. Nutrition)	29 (36)
	Knowledge Translation and Implementation	1 (<1)
	Methods	1 (<1)
	Social Welfare	29 (26)
Publication year	2017	17 (15)
	2018	11 (10)
	2019	18 (16)
	2020	11 (10)
	2021	15 (14)
	2022	20 (18)
	2023	16 (14)
	2024	3 (3)
Geographic scope^b^	Global (no specific region)	83 (75)
	OECD countries	1 (<1)
	Low‐ and middle‐income countries	23 (21)
	Latin America and the Caribbean	1 (<1)
	United States	3 (3)
Update status	Update of an earlier review	11 (10)
	Not an update	100 (90)

^a^ Nine reviews belong to more than one Coordinating Group.

^b^ Percentages may not sum to 100 because of rounding.

#### 5.1.3 Excluded studies

Any papers published in *Campbell Systematic Reviews* since 2017 that were not systematic reviews were excluded upon initial screening of tables of contents. In addition, upon data extraction we identified ten other papers that were ultimately excluded. Two studies were updates that did not change their search methods from the original reviews, which were published prior to 2017. Four studies referred to the search strategies used to assemble the Global Policing Database and did not conduct original searches themselves. Four studies used searches that were previously conducted as part of an evidence and gap map. For a list of excluded studies and their reasons for exclusion, see Table S1 in File S3.

### 5.2 Risk of bias in included studies

In assessing the search methods against Item 4 of AMSTAR 2 (Shea et al., 2017), we found that 36.0% of reviews fully met AMSTAR 2 criteria, with 48.6% meeting sufficient criteria for a ‘Partial Yes’ rating, and 23.4% receiving a ‘No’ rating. Table S2 in File S3 shows the number and percent of reviews meeting each AMSTAR 2 Item 4 sub‐criteria and overall ratings.

#### 5.2.1 Allocation

Not applicable.

#### 5.2.2 Blinding

Not applicable.

#### 5.2.3 Incomplete outcome data

Not applicable.

#### 5.2.4 Selective reporting

Not applicable.

#### 5.2.5 Other potential sources of bias

Not applicable.

### 5.3 Effects of interventions

#### 5.3.1 Source selection and reporting

**Figure 1:**
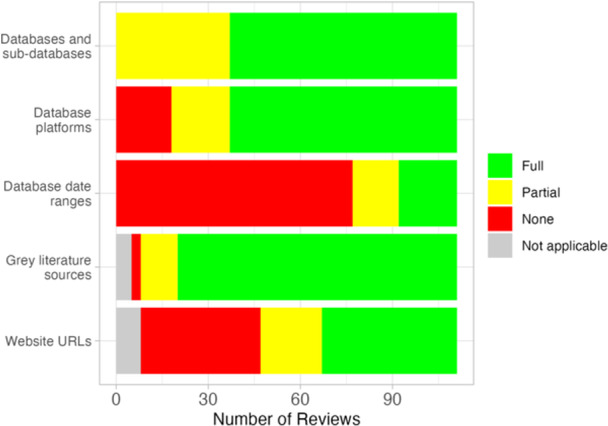
Number of reviews fully, partially or not adhering to standards related to reporting databases, grey literature and websites searched (MECCIR R34 and PRISMA Item #6).

Figure 1 summarizes reporting on sources searched. These reporting items align with mandatory reporting standards in MECCIR (standard R34) and PRISMA (Item #6). Almost all reviews searched at least two relevant databases (98.2%, *n* = 109). Of the two that did not search at least two relevant databases, one relied heavily on citation searching techniques and an existing repository of relevant literature that uses comprehensive methods to identify studies (3ie Development Evidence Portal, https://developmentevidence.3ieimpact.org/, Study ID 35). The other searched only one multidisciplinary database, and relied more on working paper repositories (Study ID 5). All reviews reported the databases searched, although 37 (33.3%) listed either an umbrella database or a platform name rather than the individual database or sub‐database name for at least one source. Platforms can host multiple databases, so listing a platform in lieu of a database makes it impossible to determine which database(s) on that platform was searched. On the other hand, listing both platform and the database name provides increased transparency and reproducibility as databases can often be accessed through multiple platforms impacting how searches are constructed (Rethlefsen et al., 2021). Eighteen reviews (16.2%) did not report any platforms along with the names of the databases. Nineteen (17.1%) reported date ranges of the contents in all of the databases searched, with 15 reporting date ranges for at least some of the databases. A heatmap of all included studies (by ID) and their degree of compliance (full, partial, none) with each data extraction item by conduct and reporting standard is shown in Figure S1 (conduct) and Figure S2 (reporting) in File S3.

With regard to web searching, a little more than half of the reviews (*n* = 60) searched Google Scholar but none of the other large free, multidisciplinary academic search engines were mentioned in any review (e.g., Dimensions, Microsoft Academic, Lens.org, etc.). A little more than one‐third of reviews reported searching Google (*n* = 42). Bing was searched in three reviews. Six reviews did not search Google, Google Scholar or any government or organizational websites. About 37.1% (*n* = 39) of reviews that reported searching websites for additional studies did not provide website URLs in either the text or supplementary material.

**Figure 2:**
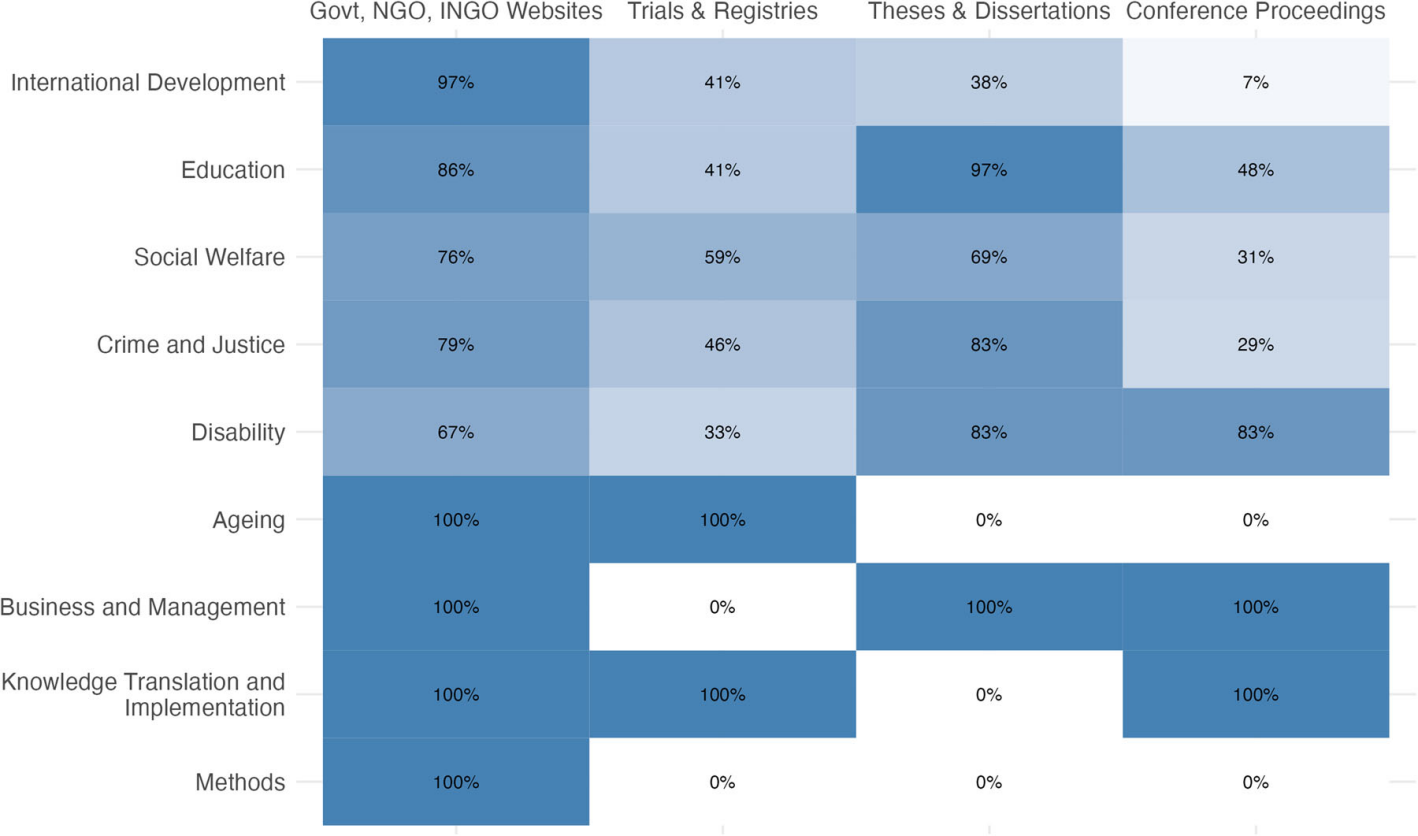
The proportion of reviews by coordinating group that intentionally searched sources of different types of grey literature (e.g., searched a dissertations and theses database as opposed to relying on coverage of dissertations and theses in the other bibliographic databases searched).

All reviews searched at least some grey literature (MECCIR C28) and most reviews (*n* = 91) provided a list of all sources of grey literature searched (MECCIR R34). Figure 2 shows the types of grey literature searched by different Campbell coordinating groups. About half of the reviews searched scholarly databases specializing in regionally specific scholarship (e.g., LILACs, Africa Journals Online, etc.).

#### 5.3.2 Search strategy conduct and reporting

**Figure 3:**
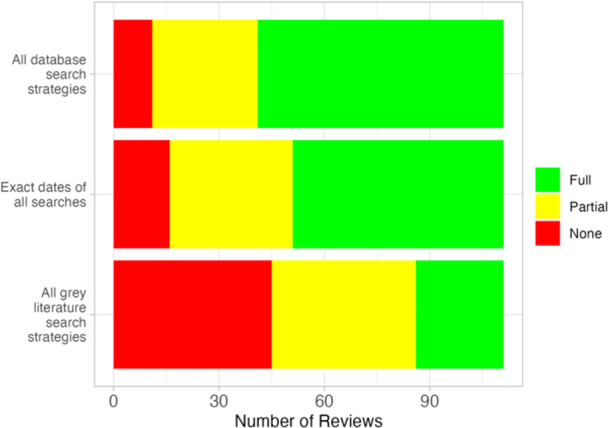
Number of reviews fully, partially or not adhering to standards related to reporting of search strategies (MECCIR R35, R38 and R39 and PRISMA Items #6,7).

Figure 3 summarizes the reporting of search strategies in Campbell systematic reviews. Most reviews reported at least one full database search strategy (90.1%, *n* = 100), with 70 (63.1%) providing full search strategies for all databases (MECCIR R38, PRISMA Item #7). Exact search dates (MECCIR R35, PRISMA Item #6) for all searches were given in 60 (54.1%) reviews, with 35 (31.5%) reporting general date ranges for search and 16 (14.4%) reporting no dates. Twenty‐five (22.5%) reviews reported all exact search strategies for grey literature sources, with 40 (36.0%) reporting some grey literature searches or a general grey literature approach, and 45 (40.5%) reporting no information about grey literature searches (MECCIR R39).

With regard to searching Google Scholar, 29 out of the 60 that used it reported a full search strategy, thirteen of which also provided details about how results were selected or screened (e.g., number of pages screened, etc.). Nine reviews provided a list of search terms used in lieu of an exact search strategy for Google Scholar, and 18 gave no details about how the Google Scholar search was conducted.

Most reviews reported search strategies that used Boolean operators (90.0%), database syntax (83.8%), and phrase searching (80.2%) correctly. Most reviews (89.2%) also used appropriate variations on keywords, such as using stemming and spelling variations. Only a little over a half (58.6%) used specific subject headings when searching databases with controlled vocabulary.

For those reviews that indicated using some database limit or filter (such as a limit on publication date range or language), 75.8% provided a justification for the use of those limits (MECCIR C35, R36). No reviews indicated that peer review had been conducted on the search strategies prior to running searches.

**Figure 4:**
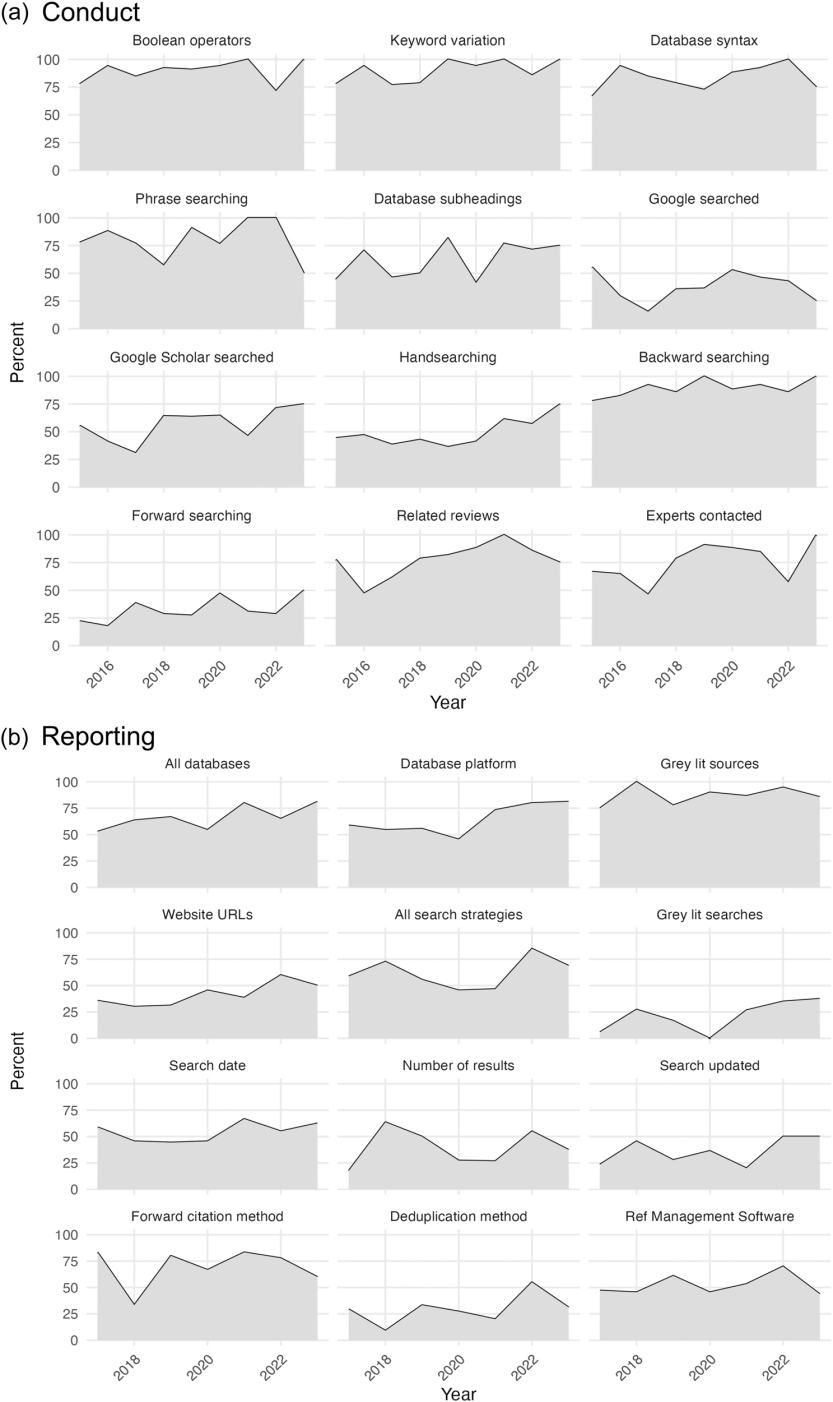
Percentage of reviews following recommended practices over time. (a) Shows variables related to the conduct of searches and search methods. Dates refer to the date the most recent search was run. Reviews with dates before 2015 and after 2024 were removed due to low numbers. (b) Shows the variables related to the reporting of searches excluding reviews published in 2024 due to low numbers. Dates refer to the date of publication.

The Campbell Collaboration published updated searching and information retrieval guidance in 2017 (Kugley et al., 2017). Of the 111 systematic reviews published since January 2017, 75 of those reviews conducted the searches after the publication of the 2017 guidance, according to the search dates provided. However, very few reviews (*n* = 12) referenced the Campbell Collaboration's information retrieval guidance document. To assess if conduct or reporting practices have improved over time, or since the publication of the 2017 guidance document, we charted the percent of reviews fully complying with each variable over time (Figure 4). This reveals no clear trends and little apparent impact of the guidance document, which aligns with its lack of citation. Note that searching guidance for Campbell did exist prior to 2017 (Hammerstrøm et al., 2010), but we did not collect data on the citation of this document in the Campbell systematic reviews we assessed.

Very few reviews explicitly indicated the use of a validated search hedge (8.1%, *n* = 9), while more (12.6%, *n* = 14) indicated that they had adapted a search strategy from a previously published review.

#### 5.3.3 Supplementary search methods

**Figure 5:**
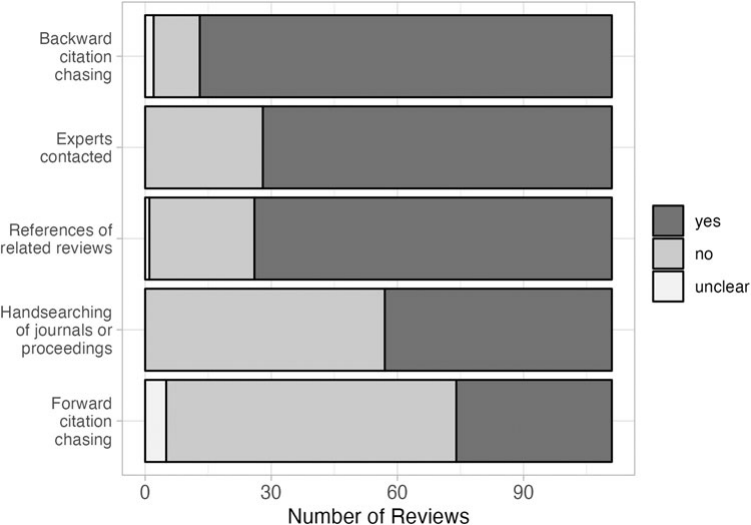
Supplementary searching methods. The number of reviews reporting the conduct of various supplementary methods typically recommended for systematic reviews.

In addition to searching sources directly (i.e., database, websites, etc.), additional methods are recommended for finding unpublished or otherwise missed studies (Papaioannou et al., 2010). This includes handsearching of journal tables of contents, contacting experts, forward citation chasing, backward citation chasing, and searching for studies in related reviews (the latter two of which are mandatory according to MECCIR conduct standards, C29 and C30). While almost all reviews used at least one of these methods, over half did not conduct any handsearching (51.4%, *n* = 57) or forward citation searching (62.2%, *n* = 69), and about one‐quarter did not report searching references of relevant reviews (22.5%, *n* = 25) or contacting experts (25.2%, *n* = 28) (Figure 5). Of those that did conduct handsearching, this was mostly of journal tables of contents as opposed to conference proceedings and the date ranges of handsearched journals were reported in 57.4% of those reviews. Only four reviews reported using listservs to solicit additional studies and input from experts, a strategy described by Kugley et al., 2017 as a potential way to identify unpublished studies.

#### 5.3.4 Involvement of information specialists

**Figure 6:**
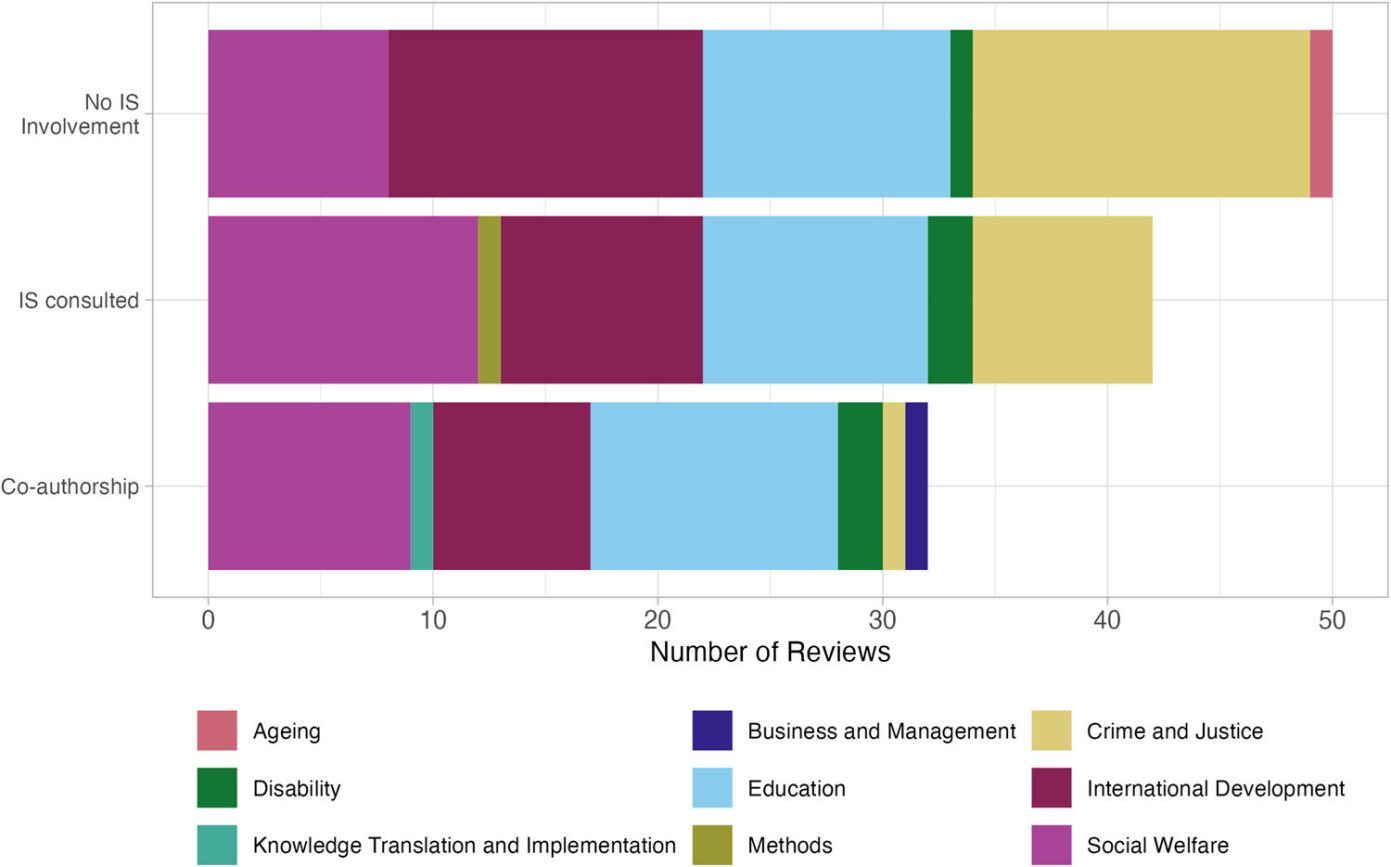
Involvement of information specialists. The number of reviews with an information specialist as a co‐author, in a consulting role only (i.e., mentioned elsewhere in the paper), or with no IS involvement mentioned. For co‐authorship, we discerned to the best of our ability whether or not any authors were librarians or information specialists (as opposed to domain experts or other methodologists). Breakdown by coordinating group is shown (using first coordinating group listed in cases where reviews are cross‐listed for more than one, for simplicity).

Information specialists (IS) or librarians trained in evidence synthesis methods can play an important supportive role in Campbell systematic reviews (Kugley et al., 2017). The degree of IS involvement in conducting searches and how they are acknowledged for their contributions varies. We found that about 45% of reviews (*n* = 50) did not mention any IS involvement. Twenty‐eight percent (*n* = 32) involved an IS as a co‐author and 37.8% (*n* = 42) involved an IS in some sort of consulting capacity (Figure 6). IS involvement was also acknowledged in different ways, for example in the Roles of Authors section (*n* = 38), methods section (*n* = 45) and acknowledgements section (*n* = 15) of reviews.

**Figure 7:**
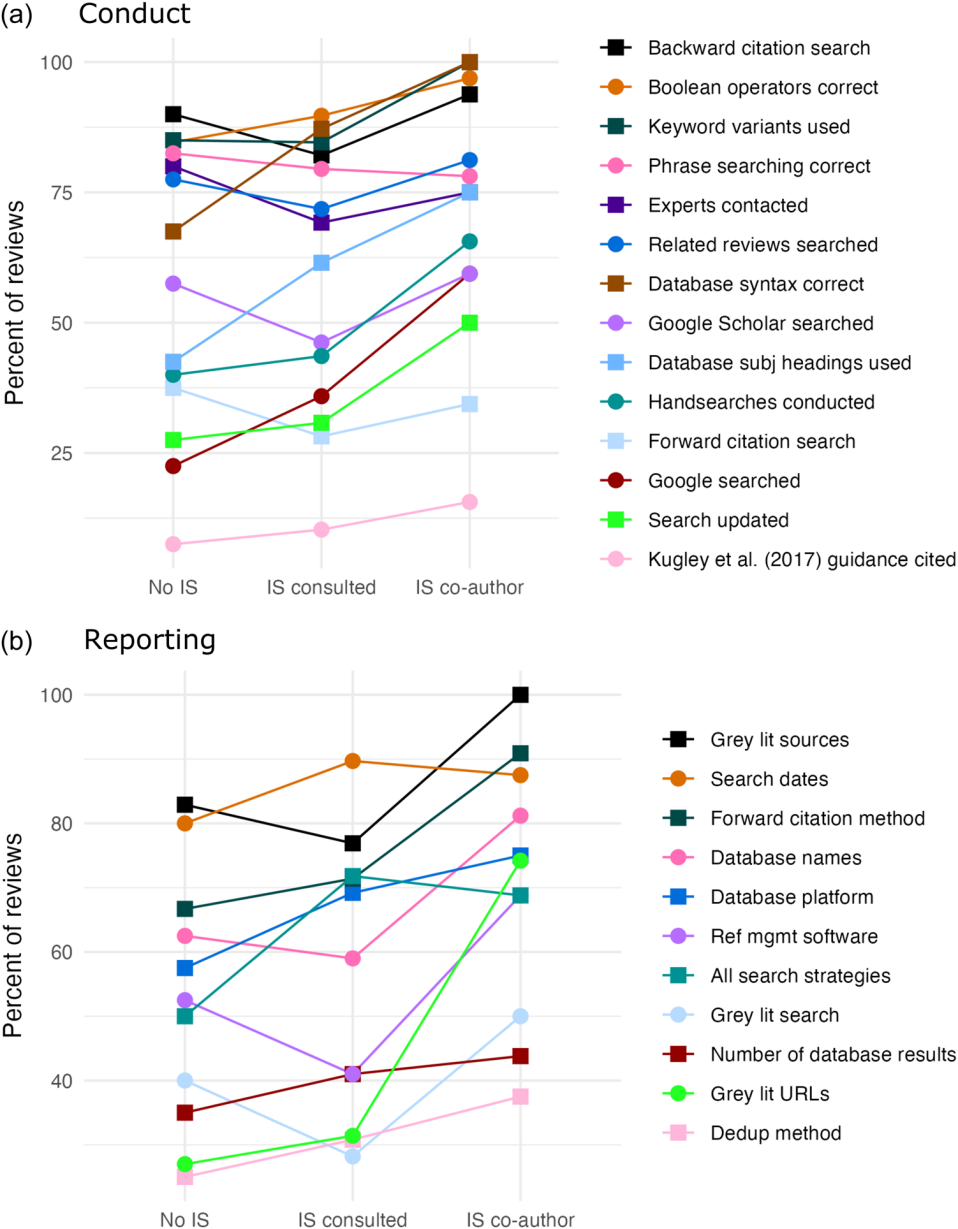
Quality of search conduct and reporting with and without information specialist involvement. Percent of reviews with no IS involvement, with indications of consultations only, and with co‐authorship in relation to compliance with (a) conduct and (b) reporting best practices. For actual numbers and percentages of adherence to search conduct and reporting standards by IS involvement, see Tables S3 and S4 in File S3.

Figure 7 shows the percentage of reviews complying with different conduct and reporting variables by level of IS involvement. The general trends indicate that those reviews with an IS co‐author are more likely to comply with different conduct and reporting practices. Among reporting elements in particular, the charts indicate more frequent reporting of grey literature searches, website URLs, methods for forward citation searching, and information management methods in reviews with IS co‐authors. For conduct variables, database syntax was used correctly for 100% of the reviews with an IS co‐author versus 67.5% of the reviews with no indicated IS involvement. Similarly, database‐specific subject headings tend to be used more often in reviews with IS co‐authors (75.0% vs. 42.5%). For the numbers and percentages underlying Figure 7, see Table S3 and S4 in File S3.

#### 5.3.5 Other considerations

##### Information management and deduplication

About half of the reviews (53.2%, *n* = 59) reported the use of reference management software for handling database search results. The most commonly mentioned reference management software applications were EndNote (*n* = 27) and EPPI‐Reviewer (*n* = 16). Other tools mentioned included Covidence (*n* = 4), Mendeley (*n* = 4), Zotero (*n* = 5), Reference Manager (*n* = 2) and Excel (*n* = 1).

Thirty‐four (30.6%) reviews reported some detail about how deduplication across sources was carried out.

##### Timeliness of search

**Figure 8:**
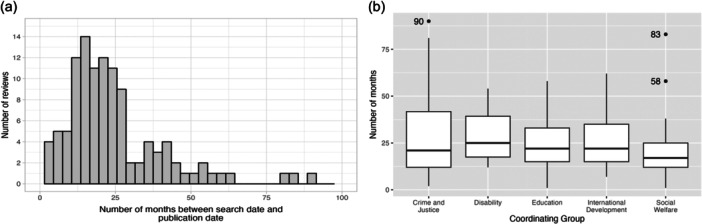
Time lag between search and publication date. (a) Histogram of the number of months elapsing between the most recent search and the date of review publication. (b) Box plot of the time lag between search and publication date by coordinating group. Coordinating groups with only one review have been excluded from the figure.

About one‐third of reviews noted that the search was updated during the course of the review before publication (35.1%, *n* = 39). Figure 8 summarizes the data about the time lag between the most recent search date and the publication date. The median number of months between the running of the most recent search and review publication was 20 (min = 1, max = 90). We used a one‐way analysis of variance (ANOVA) to compare time lag from running the search to publication date between coordinating groups and found no statistical difference between groups (*F*(4, 112) = [0.7961], *p* = 0.53; Figure 8b). MECCIR Conduct standard C37 suggests that searches should be updated within 12 months before publication of the review.

## 6 DISCUSSION

### 6.1 Summary of main results

The current work provides the first in‐depth assessment of search conduct and reporting in Campbell systematic reviews across coordinating groups. The 2019 Campbell Collaboration's conduct and reporting standards (Methods Group of the Campbell Collaboration, 2019a; 2019b) described mandatory and highly recommended items to ensure robust and transparent searches, which underlie the comprehensive and rigorous nature of these reviews. To what degree authors adhere to these standards has been unclear and this work aims to address that gap. We also sought to identify current practices around source selection, information management, search updating, and information specialist involvement.

#### 6.1.1 Source selection and reporting

We found that Campbell reviews search a wide array of sources including multiple bibliographic databases as well as specific sources of grey literature. Lists of databases were routinely reported and database platforms were reported in most cases, which is an important element of reproducibility (Rethlefsen et al., 2021). One element not well reported in Campbell reviews is database dates of coverage when available, which was also found by the assessment by Ramirez et al., 2022 of Education coordinating group reviews. While this is a mandatory reporting item in both the 2019 MECCIR and 2020 PRISMA standards, this item is not included in PRISMA‐S. Date ranges of some databases can vary by library subscription, and for reviews with no date restrictions, this information can be helpful to a reader in determining the date range of the search.

Searching grey literature is important for identifying unpublished or ongoing work (Paez, 2017). For reviews in the social sciences, grey literature can be an important source of relevant studies conducted by organizations and government agencies. We found that all Campbell reviews search for grey literature to some degree. However, reporting of the sources of grey literature searched was less consistent in its adherence to best practices and standards than database searches. While grey literature sources were listed in most reviews, URLs of websites were often unreported. Reporting of URLs is a relatively low hanging fruit that could improve transparency of grey literature searching, and could be remedied by providing author templates for search reporting.

#### 6.1.2 Search strategy conduct and reporting

The reporting of exact search strategies is critical to transparency and reproducibility of systematic reviews. Full search strategy reporting for all sources is a mandatory reporting item for both the 2019 MECCIR and PRISMA 2020 standards. Nonetheless, we found that less than 65% of Campbell reviews fully reported database searches or exact search dates. Exact search strategies for grey literature sources were far less common, with only 22.5% of reviews reporting all complete grey literature searches. Reporting of exact grey literature searches is an onerous task due to the poor search capability of many grey literature sources and the need for iteration and multiple search strategies. It requires a well‐thought out approach and a plan for documentation. Guidance and training for Campbell authors could be provided to improve grey literature search methods, documentation and reporting.

Google and Google Scholar were used in many reviews, but similarly to grey literature, search strategies and approaches for screening results were underreported. There are many challenges associated with using these platforms for systematic reviews (Gusenbauer and Haddaway, 2020). Yet, there are methods that can sufficiently capture some level of transparency in how they are used (see Briscoe and Rogers, 2021 for approaches to searching Google and a recent update by Briscoe et al., 2024). Again, more explicit guidance for Campbell authors, reporting templates and training can help address this shortcoming.

While we did not assess search quality, we did examine at a high level the use of database syntax, keywords, phrase searching and database subject headings. In general, reported searches in Campbell reviews showed sufficient use of Boolean operators, keyword variations, phrase searching, and database syntax. Subject headings in databases with controlled vocabulary were used less frequently. Understanding the nuances of different database platforms and how to most effectively combine keyword searching with controlled vocabulary is a specialty of information specialists and librarians, and thus their involvement is critical. When an information specialist is not available to collaborate with an author team, for example in resource‐limited settings without libraries and library staff, review by trained information specialists can help mitigate errors and problems in the search before searches are run. For example, a 2018 study by Spry and Mierzwinski‐Urban, 2018 found that search strategy peer review can result in additionally retrieved studies in rapid reviews. While Campbell reviews do go through a routine peer review process by an information specialist, no Campbell reviews reported peer review of search strategies outside of that standard process. Yet, this additional step can be a useful way for review teams to strengthen their protocols prior to submission and identify significant biases in their search early in the process. Moreover, after protocol review, which only requires a search strategy for the main database, the search must be adapted across many sources prior to running it. This is an entry point for potential errors, and a search peer review after search adaptation but prior to running the search can mitigate such problems. Frameworks like PRESS (McGowan et al., 2016) can be a useful tool for this added layer of peer review. That said, peer review is not a replacement for information specialist involvement in designing and running searches, as described in more detail below.

It should be noted that the search guidance document originally published in 2017 by Kugley et al., 2017 is currently, as of the writing of this manuscript, undergoing an update. We found a surprising lack of reference to this 2017 document in the reviews we looked at. This document contains detailed guidance for the conduct and reporting of searches, as well as useful lists of sources for different topic areas covered by Campbell, and yet has apparently been underutilized by Campbell authors. With its update, we recommend efforts to increase visibility to authors and other outreach to improve uptake and knowledge translation of this guidance. This could go hand in hand with promotion of the new Campbell training course for systematic reviews and meta‐analysis (Valentine et al., 2022). This online self‐paced course includes a detailed unit on searching, and could help improve search conduct and reporting particularly when author teams lack information specialist co‐authorship.

#### 6.1.3 Supplementary search methods

The use of additional methods beyond database and grey literature searching can help surface unpublished or ongoing work, or studies not found by other means for various reasons. A recent scoping review has assessed citation chasing practices in systematic reviews. They found that of the 47 studies assessing citation chasing in evidence synthesis, 96% found added value for this approach (Hirt et al., 2023). Backward citation chaining (i.e., looking through references of included studies) is required by MECCIR, and commonly conducted in Campbell reviews. Forward citation chaining may also be useful for identifying new studies but is far less common in Campbell reviews. More research is needed to determine the added value of forward citation chaining for Campbell reviews.

Handsearching is another method typically recommended for systematic reviews, and yet conducted by only about half of the included Campbell reviews published since 2017. Handsearching can be a time‐consuming and labour‐intensive task (Cooper et al., 2020), however, new tools like Paperfetcher can make this task more efficient (Pallath and Zhang, 2023). In addition, clear guidelines about how to conduct handsearching (i.e., how many back issues to review, etc.) can be difficult to find. Moreover, the added value of handsearching for Campbell reviews is not clear.

#### 6.1.4 Role of information specialists

Studies have demonstrated that the involvement of information specialists in systematic reviews improves search reporting (Aamodt et al., 2019; Meert et al., 2016; Pawliuk et al., 2024; Rethlefsen et al., 2015). Moreover, a recent study by Ramirez et al., 2022 examined adherence to published protocols of Campbell reviews from the Education Coordinating Group, and found that while overall adherence was good, it was better when reviews reported librarian involvement. We found similar trends across most conduct and reporting variables assessed as indicated by Figure 7 showing a general increase in adherence to many of these standards when an information specialist is a co‐author compared to when an information specialist is not mentioned or only consulted. In particular, there was better reporting of sources and searches in information specialist‐coauthored Campbell reviews.

While involving an information specialist in Campbell reviews is not required, it is highly recommended across Campbell's guidance and training (Kugley et al., 2017; Valentine et al., 2022; Wilson et al., 2023). Moreover, all Campbell protocols and reviews are peer reviewed by an information specialist prior to publication. Not all author teams have access to trained librarians and information specialists. Support from Campbell editorial teams could help increase information specialist involvement, in terms of consultations or pre‐submission peer reviews. Figure 6 indicates some differences across coordinating groups with respect to information specialist involvement. Working with individual coordinating groups on their workflows and author support approaches could help to develop group‐specific information specialist support to address access and use of information specialist expertise. This in turn could improve the quality of conduct and reporting of search methods.

#### 6.1.5 Other considerations

In addition to general conduct and reporting of searches, we were interested in determining the degree of time lag between the most updated search for a review and its publication. Systematic reviews can quickly become outdated with the pace of research publishing ever increasing, and the lack of an updated search strategy during the review process can exacerbate this problem. In fact, across all coordinating groups, we found a median time lag of nearly 2 years (20 months) between the most recent search and publication date, although this is within the 24 months recommended by the AMSTAR critical appraisal tool (Shea et al., 2017). The 2019 MECCIR standards highly recommend that searches be rerun within 12 months of the publication of the reviews (Methods Group of the Campbell Collaboration, 2019a), and this is a mandatory item for Cochrane reviews (Higgins et al., 2023). The length of time for peer review of Campbell Collaboration systematic reviews may contribute to this outdatedness, however we do not have data to support this idea. The current challenges in academic publishing broadly related to finding peer reviewers may exacerbate this problem (Flaherty, 2022). Workflows or guidance that encourage authors to work on updating searches during the peer review process may help to mitigate these time lags.

### 6.2 Overall completeness and applicability of evidence

This assessment includes all Campbell systematic reviews published since 2017 until the date of our search in March 2024. Our goal was to examine current methods, and thus we did not look at older reviews published before the most recent version of the Campbell search methods guidance (Kugley et al., 2017). That said, the reviews published since 2017 conducted their searches as far back as 2010, and thus represent a relatively long‐term view of search methods over time. This work provides a baseline that can be used to assess the effectiveness of any initiatives taken to improve search methods and reporting in Campbell reviews moving forward. It also provides useful information to inform such initiatives, as it summarizes current practices and identifies areas for improvement.

It is important to note that standards and guidelines documents have changed over time, even over the course of this assessment and the timeframe of the included reviews. Since at the time of our search and analysis the 2019 MECCIR reporting and conduct standards were still in place, we chose to assess the included reviews against these methodological expectations. The new MECCIR standards (Wilson et al., 2023) combine expectations for reporting and conduct into a single document and checklist and greatly simplify the requirements for reviews. Thus, there are now fewer detailed checklist items and instead, broad requirements for reproducibility and comprehensiveness. Notwithstanding, to meet these standards of reproducibility and comprehensiveness, one must adhere to best current practices and recommendations, which can be found detailed in other standards and guidelines documents, such as PRISMA‐S. We consider PRISMA‐S (Rethlefsen et al., 2021) to be the gold standard for reporting of reproducible searches, and this guided much of the assessment in the current study. In other words, despite changes to standards documents, the conduct and reporting expectations for achieving high quality, reproducible searches have remained largely unchanged.

Similarly, we chose to use the most up to date PRISMA guidance published in 2020, even though this checklist was not available to the authors at the time of many of the included studies in our assessment, as this document reflects the most current and widely used standard for reporting of systematic reviews.

### 6.3 Quality of the evidence

Not applicable.

### 6.4 Potential biases in the review process

To mitigate biases in the data extraction and assessment process, all data was extracted in duplicate with conflicts resolved by a third reviewer. All of the authors on this manuscript are information specialists involved in conducting and peer reviewing systematic reviews, and in some cases, Campbell systematic reviews. To minimize bias in the assessment process, authors with a conflict of interest for a given review (i.e., they had contributed as an author or consultant to an included review) were not involved in the data extraction for that review.

### 6.5 Agreements and disagreements with other studies or reviews

Two previous studies have examined search reporting in Campbell reviews specifically. Wang et al., 2021 assessed a number of search‐related criteria in their review based on PRISMA and AMSTAR 2, namely the reporting of sources searched, reporting of full electronic search strategies and AMSTAR 2's criteria for a comprehensive search strategy. The current work has taken a more nuanced look at these criteria, which may explain the differences in findings with Wang et al. For example, while Wang et al. reported 100% reporting of sources, we found some inadequacies in regards to reporting both the database name and platform, a detail that can impact the reproducibility of a search. Furthermore, our AMSTAR 2 analysis differs considerably from Wang et al.‘s reported findings in their Figure 1. We found that 36.0% of reviews fully met the AMSTAR 2 search criteria, while 48.6% partially met the criteria, compared to Wang et al.‘s approximately 97% fully meeting this criteria. However, based on the extracted data providing in Wang et al.'s supplementary files, our data are largely in agreement where we extracted similar data on search reporting. More details on how Wang et al. aligned search characteristics to the AMSTAR 2 checklist would be needed to determine the origin of this discrepancy.

Wang et al., 2021 also found indications that the quality of reviews improved before and after the introduction of the MECCIR standards. Our visual inspection of trends over time in search conduct and reporting did not reveal a noticeable temporal pattern. These differences could be related to overall quality vs quality related to searching specifically, or the differences in data sets with our study spanning 2017 to 2024, and Wang et al. spanning 2011 to 2018.

The second study that has examined the search characteristics of Campbell reviews is Ramirez et al., 2022. This study looked specifically at Education Coordinating Group reviews from 2014 to 2019. While our study spans all coordinating groups, many of the findings are similar including findings related to both search conduct and reporting. In addition, Ramirez et al. found indications that librarian involvement increases adherence to MECCIR standards, which aligns with our findings.

We did not conduct a comparison of Campbell systematic reviews with non‐Campbell systematic reviews. However, based on previous work of a random sample of systematic reviews indexed in Medline, Campbell reviews appear to do considerably better in terms of search conduct than non‐Campbell reviews (e.g., fewer systematic errors in the use of Boolean operators, search syntax and phrase searching) (Salvador‐Oliván et al., 2019). The quality of search conduct and reporting in Campbell reviews is more in line with what has been found previously for Cochrane reviews (see Yoshii et al., 2009 and Franco et al., 2018).

## 7 AUTHORS’ CONCLUSIONS

### 7.1 Implications for practice

Overall, our study indicates that authors of Campbell systematic reviews conduct comprehensive searches across numerous databases and sources, including grey literature and supplementary search methods, and most report full search strategies for one or more databases. However, we found that many reviews fall short of complete adherence to standards and current best practices related to the conduct and reporting of searches in systematic reviews. Some of these shortcomings are simple gaps in reporting–for example, the URLs of websites, database platforms and date ranges, and exact dates of searches–that could be addressed as long as authors are recording and documenting searches appropriately. The development of additional checklists or templates may help authors track and report these basic aspects of the search.

On the other hand, some best practices require advanced knowledge of searching. For example, while it is not required that reviewers use the controlled vocabulary of well‐indexed databases, the use of this database feature in combination with keywords can improve the comprehensiveness and quality of search strategies (Lefebvre et al., 2023). We found that reviews co‐authored by an information specialist were more likely to use controlled vocabulary. Thus, involving an information specialist in reviews may help address shortcomings in some of the more advanced aspects of searching.

Supplementary searching varied considerably across reviews, ranging from backward citation searching only to a range of techniques including forward citation searching, handsearching, searching references of reviews and contacting experts and listservs. Current guidance and recommendations suggest that all of these approaches should be applied for comprehensiveness, though this was somewhat rare amongst the reviews we looked at. More research is needed to understand the value‐add of these techniques in Campbell reviews and the implications of not using supplementary search methods.

We found that very few Campbell reviews published to date reported the use of automation, AI/ML or text mining tools in the development and execution of searches. Only one review mentioned the use of term frequency analysis for keyword term identification (Study ID 7). This may reflect the relatively nascent nature of these tools and technologies in this context. As accessibility, ease of use and trust in such tools evolves, we are likely to see an increased usage of these tools for keyword term harvesting, Boolean search construction or other aspects of the search. As guidance documents and standards evolve and are updated, they should take into consideration these emerging practices.

In summary, additional support for authors in the form of guidance, templates and training, as well as connecting author teams to librarians and information specialists with experience in evidence synthesis, may help address some of the issues identified in the current study that impact the comprehensiveness, quality and reproducibility of searches in Campbell reviews. Moreover, this study serves as a baseline to assess changes in search conduct and reporting over time and the impact of updated guidance and other initiatives to improve the quality and robustness of Campbell systematic reviews.

### 7.2 Implications for research

Included above.

## CONTRIBUTIONS OF AUTHORS

- Content: Sarah Young, Zahra Premji, Heather MacDonald, Morwenna Rogers, Alison Bethel, Ursula Ellis, Diana Louden, David Pickup
- Systematic review methods: Sarah Young, Zahra Premji, Heather MacDonald, Morwenna Rogers, Alison Bethel, Ursula Ellis, Diana Louden, David Pickup
- Data extraction: Sarah Young, Heather MacDonald, Ursula Ellis, Diana Louden, Morwenna Rogers, Alison Bethel, Zahra Premji, David Pickup
- Data analysis: Sarah Young, Heather MacDonald
- Information retrieval: Sarah Young

## DECLARATIONS OF INTEREST

Two authors (S. Y., A. B.) are currently serving as co‐conveners of the Campbell Collaboration Information Retrieval Methods Group. Other authors (S. Y., H. M., M. R., A. B., D. P., Z. P.) currently serve or have served as dedicated Campbell Collaboration coordinating group information specialists. Several authors have served as co‐authors on Campbell Collaboration reviews. Those authors were not involved in extracting data from systematic reviews in which they were involved.

## PLANS FOR UPDATING THIS REVIEW

Considerations will be given to conducting an update of this methods assessment five years after the date of publication.

## DIFFERENCES BETWEEN PROTOCOL AND REVIEW

During the course of the refinement of the data extraction tool and analysis of the data, we made minor changes that differ from the protocol. We added an additional objective focussed on information specialists and conducted analyses related to changes in conduct and reporting practices with varying levels of information specialist involvement. We additionally extracted and analysed information about whether authors reported URLs for websites searched, which is an expected reporting item in various standards and guidelines. Because we could not easily assess whether sub‐headings of thesaurus terms were purposefully excluded from searches, or omitted as an oversight, we removed a data extraction item related to whether subject heading term explosion was used correctly. Finally, in the course of determining the inclusion of studies we unexpectedly came across studies that did not conduct their own original searches, such as those that relied on the Global Policing Database. We chose to exclude these studies from the assessment.

## Supporting information

Supporting information.

Supporting information.

Supporting information.

## Data Availability

The cleaned and pre‐processed version of the data set is provided in the supplementary material and is provided, along with the code for data cleaning and analysis, in a public GitHub repository: https://github.com/rootsandberries/cc_search_methods.
