# Supplementary material for: Searching and reporting in Campbell Collaboration systematic reviews: A systematic assessment of current methods
Source: Campbell Syst Rev. 2024 Aug 21;20(3):e1432. doi: 10.1002/cl2.1432 (PMC11339316; doi:10.1002/cl2.1432)
Supplement: Supplementary file 3 — Supporting information. [file CL2-20-e1432-s003.pdf]

Supplementary Tables for Young, et al. (2024).

Table S1. Excluded studies and reasons for exclusion

**Reason for Exclusion**	**List of studies**
No updated search post-2017	Braga AA, Turchan B, Papachristos AV, Hureau DM. Hot spots policing of small geographic areas effects on crime. *Campbell Systematic Reviews*. 2019; 15:e1046. https://doi.org/10.1002/cl2.1046<br><br>Wilson, D. B., Feder, L., & Olaghere, A. Court-mandated interventions for individuals convicted of domestic violence: An updated Campbell systematic review. *Campbell Systematic Reviews*. 2021; 17:e1151. https://doi.org/10.1002/cl2.1151
Global Policing Database	Mazerolle L, Eggins E, Cherney A, Hine L, Higginson A, Belton E. Police programmes that seek to increase community connectedness for reducing violent extremism behaviour, attitudes and beliefs. *Campbell Systematic Reviews*. 2020; 16:e1111. https://doi.org/10.1002/cl2.1111<br><br>Lum C, Koper CS, Wilson DB, et al. Body-worn cameras' effects on police officers and citizen behavior: A systematic review. *Campbell Systematic Reviews*. 2020; 16:e1112. https://doi.org/10.1002/cl2.1112<br><br>Mazerolle L, Cherney A, Eggins E, Hine L, Higginson A. Multiagency programs with police as a partner for reducing radicalisation to violence. *Campbell Systematic Reviews*, 2021, 17, e1162. https://doi.org/10.1002/cl2.1162<br><br>Petersen, K., Davis, R. C., Weisburd, D., & Taylor, B. (2022). Effects of second responder programs on repeat incidents of family abuse: An updated systematic review and meta-analysis. *Campbell Systematic Reviews*, 18, e1217. https://doi.org/10.1002/cl2.1217
Search from evidence and gap map	Keenan, C., Miller, S., Hanratty, J., Pigott, T, Hamilton, J., Coughlan, C., Mackie, P., Fitzpatrick, S., & Cowman, J. Accommodation-based interventions for individuals experiencing, or at risk of experiencing, homelessness. *Campbell Systematic Reviews*. 2021;e1165. https://doi.org/10.1002/cl2.1165<br><br>Hunt, X., Saran, A., Banks, L. M., White, H., & Kuper, H. (2022). Effectiveness of interventions for improving livelihood outcomes for people with disabilities in low- and middle-income countries: A systematic review. *Campbell Systematic Reviews*, 18, e1257. https://doi.org/10.1002/cl2.1257<br><br>Jain, M., Shisler, S., Lane, C., Bagai, A., Brown, E., Engelbert, M., Vardy, Y., Eyers, J., Leon, D. A., & Parsekar, S. S. (2022). Use of community engagement interventions to improve child immunisation in low- and middle-income countries: A systematic review

	and meta-analysis. *Campbell Systematic Reviews*, 18, e1253. https://doi.org/10.1002/cl2.1253<br><br>Whear, R., Campbell, F., Rogers, M., Sutton, A., Robinson-Carter, E., Sharpe, R., Cohen, S., Fergy, R., Garside, R., Kneale, D., Melendez-Torres, G. J., & Thompson-Coon, J. (2023). What is the effect of intergenerational activities on the wellbeing and mental health of older people?: A systematic review. *Campbell Systematic Reviews*, 19, e1355. https://doi.org/10.1002/cl2.1355

Supplementary Tables for Young, et al. (2024).

Table S2. The number and percent of reviews meeting the criteria in Item #4 of the AMSTAR 2 critical appraisal tool, "Did the review authors use a comprehensive literature search strategy?". The last three rows indicate whether reviews fully met (Yes), only partially met (Partial Yes) or did not meet (No) the AMSTAR 2 criteria for a comprehensive search. Note that because not all Campbell reviews would be expected to search trial registries, this criteria was not included in the overall assessment.

	Meets AMSTAR 2 criteria	
	Number of reviews	Percent of reviews (%)
Searched at least 2 databases	109	98.2
Provided search strategy	100	90.1
Justified publication restrictions	95	85.6
Searched the reference lists of included studies	98	88.3
Searched trial/study registries	53	47.7
Included/consulted content experts	83	74.8
Searched for grey literature	106	95.5
Conducted search within 24 months	70	63.1
**Yes**	**40**	**36.0**
**Partial Yes**	**54**	**48.6**
**No**	**26**	**23.4**

Table S3. Role of information specialists (IS) and adherence to conduct standards and guidelines.

	Conduct of searches					
	No IS Involvement (n=37)		IS consulted (n=42)		IS co-author (n=32)	
	No.	%	No.	%	No.	%
Boolean operators used correctly	33	84.6	35	89.7	31	96.9
Database subj heading/thesauri used	17	42.5	24	61.5	24	75.0
Keyword variants used	34	85.0	33	84.6	32	100.0
Phrase searching used correctly	33	82.5	31	79.5	25	78.1
Database syntax used correctly	27	67.5	34	87.2	32	100.0
Google Scholar searched	23	57.5	18	46.2	19	59.4
Google searched	9	22.5	14	35.9	19	59.4
Handsearches conducted	16	40.0	17	43.6	21	65.6
Experts contacted	32	80.0	27	69.2	24	75.0
Backward citation searching conducted	36	90.0	32	82.1	30	93.8
Forward citation searching conducted	15	37.5	11	28.2	11	34.4
References of related reviews searched	31	77.5	28	71.8	26	81.2
Search updated prior to publication	11	27.5	12	30.8	16	50.0
Kugley et al. (2017) guidance cited	3	7.5	4	10.3	5	15.6

Supplementary Tables for Young, et al. (2024).

Table S4. Role of information specialists (IS) and adherence to reporting standards and guidelines.

	Reporting of searches					
	No IS Involvement (n=37)		IS consulted (n=42)		IS co-author (n=32)	
	No.	%	No.	%	No.	%
All search strategies reported	20	50.0	28	71.8	22	68.8
All databases and sub-databases listed	25	62.5	23	59.0	26	81.2
Database platform reported	23	57.5	27	69.2	24	75.0
Search dates reported	32	80.0	35	89.7	28	87.5
Grey lit sources listed	29	82.9	30	76.9	32	100.0
Grey lit search reported	16	40.0	11	28.2	16	50.0
Grey lit URLs reported	10	27.0	11	31.4	23	74.2
Method for forward citation searching described	12	66.7	10	71.4	10	90.9
Reference management software reported	21	52.5	16	41.0	22	68.8
Deduplication method reported	10	25.0	12	30.8	12	37.5
Number of records per database reported	14	35.0	16	41.0	14	43.8

MECCIR	C24	C25		C27			C28						C29	C30	C31		C33				C35	C36		37/C38	
Study ID	kugley	two_db	geog_db	gray_text	trials	registries	gray_text	conf_proc	theses	govt	ngo	hand_conf	reviews	backward	experts	experts_listserv	subhead	keyword_var	phrase	syntax	limit_just	search_text	date_searches	update_search	Coordinating Group
111	0	2	0	2	2	2	0	0	0	2	2	0	2	2	2	0	2	2	2	2	NA	2	2	2	A
112	0	2	0	2	0	0	2	2	2	2	2	2	0	2	2	0	2	2	2	2	2	2	2	2	BM
10	0	2	2	2	0	2	0	0	2	2	2	0	2	2	2	0	0	2	UN	UN	NA	2	0	0	CJ
11	0	2	2	2	2	0	0	0	0	2	2	0	0	0	2	0	2	2	2	2	2	2	2	0	CJ
23	0	2	2	2	0	0	0	0	2	2	2	0	2	2	2	0	0	2	2	0	NA	2	2	0	CJ
29	0	2	0	2	2	2	2	2	2	2	2	2	2	2	2	0	0	0	2	2	NA	2	1	2	CJ
30	0	2	0	2	2	2	0	0	0	0	0	0	2	2	2	0	2	2	0	2	2	2	2	0	CJ
36	0	2	2	2	0	2	2	2	2	2	2	2	2	UN	2	0	0	0	0	0	UN	2	1	0	CJ
37	0	2	2	2	0	2	0	0	2	2	0	0	2	2	2	0	0	0	UN	0	UN	2	1	0	CJ
49	0	2	0	2	2	0	2	2	2	2	2	0	UN	2	0	0	2	2	2	2	0	2	1	0	CJ
55	0	2	2	2	0	0	2	2	2	2	2	0	0	0	2	0	UN	2	2	2	2	2	1	0	CJ
56	0	2	0	2	0	0	0	0	0	2	2	0	0	2	2	0	0	0	0	0	2	2	1	2	CJ
63	0	0	0	2	0	0	0	0	2	0	2	0	0	2	0	0	UN	UN	UN	UN	2	2	0	0	CJ
72	0	2	2	2	2	0	0	0	2	0	0	0	2	0	0	0	0	2	0	0	2	2	0	0	CJ
73	0	2	0	2	0	0	0	0	2	2	2	0	2	2	2	0	0	2	0	2	NA	2	1	0	CJ
79	0	2	2	2	0	0	2	2	0	0	0	0	0	2	2	0	0	2	2	2	NA	2	1	2	CJ
80	0	2	0	2	2	0	0	0	2	2	0	0	2	2	0	0	0	2	2	2	2	2	2	0	CJ
91	0	2	2	2	0	0	UN	UN	2	2	2	0	2	2	0	0	0	2	2	2	2	2	2	2	CJ
93	0	2	0	2	0	0	0	0	2	0	0	0	2	2	2	0	0	2	2	2	0	2	1	2	CJ
94	2	2	0	2	0	0	2	2	2	2	2	0	2	2	2	2	2	2	2	2	0	2	2	0	CJ
95	0	2	2	2	0	0	UN	UN	2	2	0	0	2	2	2	0	0	2	2	0	NA	2	1	0	CJ
102	0	2	2	2	0	0	0	0	2	2	2	0	2	2	2	0	2	2	2	2	2	2	2	0	CJ
120	2	2	2	2	2	2	UN	UN	0	2	2	0	2	2	2	0	0	2	0	0	NA	2	1	0	CJ
28	0	2	2	2	2	2	0	0	2	0	2	0	2	2	2	0	0	2	2	0	NA	2	2	0	CJ;E
18	0	2	2	2	0	0	UN	UN	2	2	2	0	0	2	2	0	2	2	2	2	NA	2	1	0	CJ;ID
38	0	2	0	2	0	0	0	0	2	0	0	0	2	2	0	0	0	0	2	0	NA	2	0	0	CJ;SW
31	0	2	2	2	0	0	2	2	2	0	0	2	2	2	2	0	2	2	2	2	2	2	1	0	D
51	0	2	0	2	0	0	UN	UN	2	2	2	0	0	2	0	0	2	2	UN	2	2	2	0	0	D
52	0	2	2	2	2	2	2	2	2	2	2	0	2	2	2	0	2	2	2	2	2	2	2	2	D

MECCIR	C24	C25		C27			C28						C29	C30	C31		C33				C35	C36		37/C38	
Study ID	kugley	two_db	geog_db	gray_text	trials	registries	gray_text	conf_proc	theses	govt	ngo	hand_conf	reviews	backward	experts	experts_listserv	subhead	keyword_var	phrase	syntax	limit_just	search_text	date_searches	update_search	Coordinating Group
103	0	2	0	2	0	0	2	2	2	2	2	0	2	2	2	0	2	2	2	2	2	2	2	0	D
113	0	2	2	2	UN	2	2	2	UN	0	0	UN	2	0	0	0	2	2	2	2	NA	2	1	0	D
4	0	2	2	2	2	2	2	2	2	2	2	UN	0	2	2	0	2	2	2	2	NA	2	2	2	E
5	0	2	0	2	0	0	UN	UN	2	0	0	0	2	0	0	0	2	2	2	2	0	2	1	2	E
8	0	2	2	2	2	0	0	0	2	0	2	0	2	2	2	0	0	0	2	2	NA	2	2	0	E
9	0	2	0	2	0	0	0	0	2	0	2	0	0	2	2	0	0	2	2	2	2	2	0	0	E
12	0	2	0	2	2	2	0	0	2	0	2	0	0	2	2	0	2	2	2	2	NA	2	2	0	E
19	0	2	0	2	0	0	0	0	2	2	2	0	2	2	0	0	0	2	2	2	0	2	2	2	E
22	0	2	0	2	2	0	2	2	2	2	2	0	2	0	2	0	0	2	2	2	2	2	0	0	E
32	2	2	2	2	0	0	UN	UN	2	0	0	0	0	0	0	0	2	2	2	2	0	2	0	0	E
33	0	2	0	2	UN	0	2	2	2	2	2	2	2	2	2	0	0	0	2	2	2	2	2	0	E
42	2	2	0	2	0	UN	0	0	2	2	2	0	0	2	0	0	2	2	2	2	2	2	2	0	E
44	0	2	0	2	0	0	2	2	2	0	0	0	2	UN	2	0	2	2	2	2	0	2	1	2	E
53	0	2	2	2	UN	2	UN	UN	2	2	2	0	2	2	2	0	2	2	UN	2	2	2	1	2	E
64	0	2	2	2	UN	2	2	2	2	0	2	0	2	2	2	0	UN	2	0	2	2	2	2	2	E
81	0	2	0	2	0	0	0	0	2	0	2	0	2	2	0	0	2	2	2	2	NA	2	0	0	E
82	0	2	0	2	UN	0	2	2	2	2	2	2	2	0	2	0	0	2	2	2	2	2	1	2	E
89	0	2	2	2	UN	0	0	0	2	0	2	0	2	2	0	0	2	2	2	2	NA	2	0	2	E
99	0	2	2	2	0	0	2	2	2	2	2	2	2	2	2	0	2	2	2	2	2	2	2	0	E
107	0	2	0	2	0	0	2	2	2	2	2	2	2	2	2	0	0	2	2	2	NA	2	1	2	E
108	0	2	0	2	0	0	UN	UN	2	2	2	0	2	2	0	0	2	2	2	2	NA	2	2	0	E
109	2	2	2	2	2	0	2	2	2	0	0	0	2	2	2	0	2	2	2	2	NA	2	1	2	E
114	0	2	2	2	2	2	2	2	2	2	2	0	2	2	2	0	2	2	2	2	NA	2	2	2	E
115	0	2	2	2	2	2	2	2	2	2	2	0	2	2	2	2	0	2	2	2	2	2	0	2	E;CJ
74	0	2	0	2	UN	0	2	2	2	0	2	2	0	2	0	0	2	2	0	2	2	2	2	0	E;D
7	0	2	0	2	0	0	2	2	2	2	2	2	2	2	2	0	2	2	2	2	2	2	2	0	E;ID;SW
15	0	2	2	2	UN	0	2	2	2	2	2	2	2	2	2	0	0	2	2	2	2	2	1	0	E;SW
20	2	2	0	2	2	2	0	0	2	2	2	0	2	2	2	0	0	2	2	2	NA	2	1	2	E;SW
24	0	2	2	2	UN	0	0	0	0	2	2	0	2	2	0	0	2	2	0	2	2	2	1	2	E;SW

MECCIR	C24	C25		C27			C28						C29	C30	C31		C33				C35	C36		37/C38	
Study ID	kugley	two_db	geog_db	gray_text	trials	registries	gray_text	conf_proc	theses	govt	ngo	hand_conf	reviews	backward	experts	experts_listserv	subhead	keyword_var	phrase	syntax	limit_just	search_text	date_searches	update_search	Coordinating Group
2	0	2	0	2	0	0	0	0	0	2	2	0	0	2	2	0	0	2	2	2	NA	2	0	0	ID
3	0	2	2	2	0	0	UN	UN	0	2	2	0	2	2	0	0	2	2	2	2	2	2	2	0	ID
6	0	2	0	2	0	0	0	0	0	2	2	0	2	2	2	0	0	2	2	2	NA	2	1	0	ID
13	0	2	2	2	0	0	0	0	2	2	2	0	0	2	2	2	2	2	2	2	2	2	2	0	ID
25	0	2	2	2	0	0	0	0	2	2	2	0	2	2	2	0	UN	2	2	2	NA	2	1	0	ID
34	2	2	0	2	0	0	0	0	0	2	2	0	2	2	2	0	2	2	2	2	2	2	2	0	ID
40	0	2	0	2	0	0	0	0	0	2	2	0	2	2	2	0	0	2	2	2	2	2	2	0	ID
41	0	2	2	2	UN	2	0	0	0	2	2	0	2	2	2	0	2	2	2	2	NA	2	2	2	ID
47	0	2	2	2	0	0	0	0	0	2	2	0	0	2	0	0	2	0	0	0	2	2	0	0	ID
48	0	2	2	2	0	0	0	0	2	2	2	0	0	2	2	0	0	2	2	2	2	2	0	0	ID
54	0	2	2	2	2	2	0	0	0	2	2	0	2	2	2	0	2	2	2	0	NA	2	2	0	ID
65	0	2	0	2	0	0	UN	UN	0	2	2	0	0	2	2	0	0	2	2	2	2	2	2	0	ID
66	0	2	2	2	2	2	UN	UN	2	2	2	0	2	2	2	0	2	2	2	2	2	2	0	0	ID
75	0	2	0	2	0	0	2	2	0	2	2	0	2	2	2	0	2	2	2	2	2	2	2	0	ID
76	0	2	0	2	0	0	0	0	2	2	2	0	0	2	2	0	0	2	2	0	2	2	2	0	ID
77	0	2	UN	2	0	0	0	0	0	2	2	0	2	2	2	0	2	2	2	2	2	2	1	0	ID
83	0	2	2	2	0	2	0	0	2	2	2	0	2	2	2	0	2	2	0	2	2	2	1	2	ID
84	0	2	2	2	0	0	0	0	0	2	2	0	2	2	2	UN	UN	2	2	2	2	2	2	0	ID
104	0	2	0	2	2	2	0	0	2	2	2	0	2	2	2	0	2	2	2	2	NA	2	2	0	ID
116	0	2	0	2	UN	0	0	0	0	2	2	0	2	2	0	0	0	0	0	UN	NA	2	0	0	ID
118	0	2	0	2	2	2	0	0	0	2	2	0	2	2	2	0	2	2	2	2	NA	2	2	0	ID
43	0	2	2	2	2	2	0	0	0	2	2	0	2	2	2	0	2	2	2	2	NA	2	2	0	ID;N
50	0	2	2	0	2	2	0	0	0	0	0	0	0	2	2	0	2	2	2	2	0	2	2	0	ID;N
61	0	2	2	2	2	2	0	0	0	2	2	0	2	2	2	0	2	2	UN	2	UN	2	2	2	ID;N
67	0	2	2	2	2	UN	0	0	2	2	2	0	2	2	2	0	2	2	2	0	NA	2	2	0	ID;N
68	0	2	2	2	2	2	0	0	2	2	2	0	2	2	2	0	2	2	2	0	NA	2	2	0	ID;N
21	0	2	0	2	2	0	2	2	0	2	2	0	2	2	0	0	2	2	2	2	NA	2	1	0	KTI
35	0	0	0	2	0	0	0	0	0	0	2	0	2	2	0	0	0	2	2	2	NA	2	1	0	M
1	0	2	2	2	2	2	UN	UN	0	0	0	0	0	2	0	0	2	2	2	2	2	2	2	0	SW

MECCIR	C24	C25		C27			C28						C29	C30	C31		C33				C35	C36		37/C38	
Study ID	kugley	two_db	geog_db	gray_text	trials	registries	gray_text	conf_proc	theses	govt	ngo	hand_conf	reviews	backward	experts	experts_listserv	subhead	keyword_var	phrase	syntax	limit_just	search_text	date_searches	update_search	Coordinating Group
14	0	2	2	2	2	2	0	0	2	0	0	0	2	2	2	0	2	2	2	2	2	2	2	2	SW
16	0	2	2	2	2	0	0	0	2	2	2	0	2	2	2	0	2	2	2	2	NA	2	2	2	SW
26	0	2	0	2	0	0	0	0	2	2	2	0	2	2	0	0	2	2	2	2	NA	2	2	2	SW
27	0	2	0	2	2	0	0	0	0	0	0	0	0	2	0	0	2	2	2	2	2	2	2	2	SW
45	0	2	0	2	0	0	0	0	0	2	2	0	2	2	0	0	0	2	0	2	2	2	2	2	SW
46	0	2	2	2	0	2	2	2	2	2	2	2	2	2	2	2	2	2	2	2	NA	2	1	2	SW
58	0	2	0	2	2	2	0	0	2	2	2	0	2	2	2	0	2	2	2	2	NA	2	2	2	SW
60	0	2	0	2	0	0	0	0	0	2	2	0	2	2	2	0	0	2	2	2	NA	2	2	0	SW
71	0	2	0	0	2	0	0	0	0	0	0	0	0	2	2	0	2	2	0	2	0	2	2	0	SW
78	0	2	2	2	2	0	0	0	2	2	0	0	2	0	2	0	2	2	2	2	2	2	2	2	SW
85	2	2	0	2	0	2	0	0	2	2	2	0	2	2	2	0	0	2	2	2	NA	2	1	2	SW
86	0	2	0	2	0	0	0	0	2	0	0	0	2	2	2	0	2	2	2	2	NA	2	2	0	SW
88	0	2	0	2	2	0	0	0	2	2	2	0	2	0	0	0	2	2	2	2	0	2	2	0	SW
90	2	2	0	2	UN	0	2	2	2	2	2	0	2	2	2	0	2	2	2	2	NA	2	2	2	SW
92	2	2	2	2	0	0	UN	UN	0	2	2	0	2	0	2	0	0	2	2	2	2	2	1	0	SW
98	0	2	0	2	0	2	2	2	0	UN	2	0	0	2	0	0	2	0	2	2	2	2	2	2	SW
100	2	2	0	2	2	2	2	2	2	2	2	2	2	2	2	0	0	2	2	2	2	2	2	2	SW
101	0	2	0	2	0	0	UN	UN	2	0	2	0	2	2	2	0	2	2	2	2	NA	2	2	0	SW
105	2	2	0	2	UN	2	2	2	2	2	2	0	2	2	2	0	2	2	2	2	NA	2	1	2	SW
106	0	2	0	2	UN	2	0	0	0	2	2	0	2	2	2	0	2	2	2	2	2	2	2	0	SW
110	0	2	2	2	2	2	2	2	2	2	2	0	2	2	2	0	0	2	2	2	NA	2	2	2	SW
119	0	2	0	2	2	0	0	0	2	UN	UN	0	2	2	2	0	2	2	2	2	0	2	2	0	SW
121	0	2	0	2	0	0	2	2	2	2	2	UN	2	2	2	0	2	2	0	2	0	2	1	0	SW
17	0	2	2	2	2	0	0	0	2	2	2	0	2	2	2	0	0	0	0	0	2	2	0	0	SW

Figure S1. Heatmap summary of adherence to conduct standards

Colours (indicate degree of agreement with variables): blue (complete - 2), yellow (partial - 1), red (none - 0), grey (unclear), white (not applicable - NA)

MECCIR	C24	C25		C27			C28						C29	C30	C31		C33				C35	C36		37/C38	
Study ID	kugley	two_db	geog_db	gray_text	trials	registries	gray_text	conf_proc	theses	govt	ngo	hand_conf	reviews	backward	experts	experts_listserv	subhead	keyword_var	phrase	syntax	limit_just	search_text	date_searches	update_search	Coordinating Group

Coordinating Groups: A (Aging): BM (Business and Management); CJ (Criminal Justice); D (Disability); E (Education); ID (International Development); N (Nutrition); KTI (Knowledge Translation and Implementation); M (Methods); SW (Social Welfare)

Standards: MECCIR (Methodological Expectations for Campbell Collaboration Intervention Reviews, 2019)

Study Variables: See Supplementary File 1: Data extraction form and codebook

	db_list	db_platform	gray_list	gray_url	registries	trials	govt	ngo	date_range	backward	date_searches	update_search	limit_date	limit_just	strategy_all	gray_search	reviews	handsearch	hand_journ	hand_conf	forward	forward_method	0	experts_listserv	limit_lang	hedge	adapt	adapt_cite	peer_review	num_records	deduplicate	Coordinating Groups
**PRISMA-S**	1,2	1	3		3	3				5	13	12	9	9	8	8	5	4	4	4	5	5	6	6	9	10	11	11	14	15	16	
**PRISMA**	6		6		6	6				6	6				7	7	6															
**MECCIR**	R34										R35		R36		R38	R39																
Study ID																																
111	2	2	2	2	2	2	2	2	0	2	2	2	0	NA	2	2	2	0	0	0	0	NA	2	0	0	0	0	NA	0	0	2	A
112	2	2	2	2	0	0	2	2	0	2	2	2	2	2	2	2	0	2	2	2	2	2	0	0	0	0	0	NA	0	2	2	BM
10	1	0	1	0	2	0	2	2	0	2	0	0	2	NA	0	2	2	2	2	0	0	NA	2	0	1	0	2	2	0	0	0	CJ
11	2	2	2	2	0	2	2	2	0	0	2	0	2	2	2	0	0	0	0	0	0	NA	2	0	0	0	2	0	0	2	2	CJ
23	1	1	2	0	0	0	2	2	0	2	2	0	0	NA	2	2	2	0	0	0	0	NA	0	0	0	0	0	NA	0	2	0	CJ
29	2	1	2	2	2	2	2	2	0	2	1	2	0	NA	0	0	2	2	2	2	2	2	2	0	0	0	0	NA	0	0	0	CJ
30	1	1	2	2	2	2	0	0	1	2	2	0	0	2	2	0	2	0	0	0	0	NA	2	0	0	2	0	NA	0	2	0	CJ
36	2	0	1	0	2	0	2	2	0	UN	1	0	UN	UN	0	0	2	2	2	2	2	2	2	0	UN	0	0	NA	0	0	0	CJ
37	1	1	2	0	2	0	2	0	0	2	1	0	UN	UN	0	2	2	2	2	0	2	0	0	0	UN	0	0	NA	0	0	0	CJ
49	1	1	2	2	0	2	2	2	1	2	1	0	0	0	0	2	UN	0	0	0	0	NA	2	0	0	0	0	NA	0	0	2	CJ
55	2	2	2	0	0	0	2	2	0	0	1	0	2	2	0	2	0	2	2	0	2	0	2	0	0	0	0	NA	0	0	0	CJ
56	1	0	2	0	0	0	2	2	0	2	1	2	2	2	0	2	0	0	0	0	0	NA	2	0	0	0	0	NA	0	0	2	CJ
63	1	2	2	0	0	0	0	2	0	2	0	0	UN	2	UN	0	0	0	0	0	2	2	2	0	1	0	0	NA	0	0	0	CJ
72	2	2	1	NA	0	2	0	0	0	0	0	0	2	2	0	0	2	0	0	0	0	NA	2	0	0	0	0	NA	0	0	0	CJ
73	2	1	2	0	0	0	2	2	0	2	1	0	0	NA	0	1	2	2	2	0	0	NA	0	0	0	0	0	NA	0	0	0	CJ
79	2	2	2	0	0	0	0	0	2	2	1	2	0	NA	2	2	0	0	0	0	0	NA	0	0	2	0	0	NA	0	0	0	CJ
80	1	2	2	2	0	2	2	0	2	2	2	0	2	2	2	2	2	2	UN	0	2	2	0	0	2	0	UN	2	0	0	2	CJ
91	1	2	2	2	0	0	2	2	0	2	2	2	2	2	2	2	2	2	2	0	2	2	2	0	2	0	2	2	0	2	0	CJ
93	2	2	NA	0	0	0	0	0	2	2	1	2	2	0	2	0	2	0	0	0	0	NA	2	0	0	0	0	NA	0	2	2	CJ
94	2	2	2	2	0	0	2	2	2	2	2	0	0	0	2	0	2	2	2	0	2	2	2	2	0	0	0	NA	0	0	2	CJ
95	2	2	2	2	0	0	2	0	2	2	1	0	0	NA	2	2	2	2	2	0	2	2	0	0	0	0	0	NA	0	0	2	CJ
102	2	2	NA	0	0	0	2	2	0	2	2	0	2	2	2	2	2	2	2	0	2	2	2	0	0	0	0	NA	0	2	0	CJ
120	1	2	1	0	2	2	2	2	0	2	1	0	0	NA	2	2	2	0	0	0	0	NA	2	0	0	2	0	NA	0	0	0	CJ
28	1	1	2	0	2	2	0	2	0	2	2	0	0	NA	2	2	2	0	0	0	0	NA	2	0	0	0	0	NA	0	2	0	CJ;E
18	1	2	2	1	0	0	2	2	1	2	1	0	0	NA	2	1	0	2	2	0	2	0	0	0	0	2	2	2	0	0	0	CJ;ID
38	2	0	2	NA	0	0	0	0	0	2	0	0	0	NA	0	2	2	0	0	0	0	NA	2	0	0	0	0	NA	0	0	2	CJ;SW
31	2	0	2	1	0	0	0	0	0	2	1	0	2	2	2	2	2	2	2	2	UN	UN	0	0	0	0	0	NA	0	2	0	D
51	1	1	2	0	0	0	2	2	0	2	0	0	2	2	0	UN	0	0	0	0	0	NA	2	0	1	0	2	2	0	0	2	D
52	2	2	2	0	2	2	2	2	0	2	2	2	2	2	2	1	2	2	2	0	0	NA	2	0	0	0	0	NA	0	0	0	D
103	2	2	2	1	0	0	2	2	2	2	2	0	2	2	2	0	2	0	0	0	2	0	2	0	0	0	2	2	0	2	2	D
113	2	2	1	0	2	UN	0	0	0	0	1	0	0	NA	2	0	2	2	2	UN	0	NA	2	0	0	0	0	NA	0	0	0	D
4	2	2	2	1	2	2	2	2	0	2	2	2	0	NA	2	0	0	2	2	UN	0	NA	0	0	0	0	0	NA	0	0	0	E
5	1	2	2	NA	0	0	0	0	0	0	1	2	2	0	2	2	2	2	2	0	0	NA	2	0	2	0	0	NA	0	0	2	E

Study ID	db_list	db_platform	gray_list	gray_url	registries	trials	govt	ngo	date_range	backward	date_searches	update_search	limit_date	limit_just	strategy_all	gray_search	reviews	handsearch	hand_journ	hand_conf	forward	forward_method	0	experts_listserv	limit_lang	hedge	adapt	adapt_cite	peer_review	num_records	deduplicate	Coordinating Groups
**PRISMA-S**	1,2	1	3		3	3				5	13	12	9	9	8	8	5	4	4	4	5	5	6	6	9	10	11	11	14	15	16	
**PRISMA**	6		6		6	6				6	6				7	7	6															
**MECCIR**	R34										R35		R36		R38	R39																
8	2	2	2	2	0	2	0	2	1	2	2	0	0	NA	2	1	2	2	2	0	2	2	2	0	0	0	0	NA	0	2	0	E
9	2	2	1	0	0	0	0	2	0	2	0	0	2	2	2	0	0	0	0	0	0	NA	2	0	0	0	0	NA	0	0	0	E
12	1	1	NA	NA	2	2	0	2	0	2	2	0	0	NA	2	0	0	0	0	0	0	NA	2	0	0	0	0	NA	0	0	0	E
19	2	2	2	0	0	0	2	2	0	2	2	2	2	0	0	0	2	2	2	0	0	NA	2	0	0	0	0	NA	0	0	0	E
22	2	2	2	0	0	2	2	2	0	0	0	0	2	2	2	2	2	2	2	0	0	NA	2	0	0	0	0	NA	0	2	0	E
32	1	2	2	NA	0	0	0	0	0	0	0	0	2	0	0	0	0	0	0	0	0	NA	2	0	0	0	0	NA	0	0	0	E
33	2	0	2	1	0	UN	2	2	0	2	2	0	2	2	2	0	2	2	0	2	0	NA	2	0	0	0	0	NA	0	0	2	E
42	1	2	0	0	UN	0	2	2	0	2	2	0	2	2	2	0	0	0	0	0	2	2	2	0	0	0	2	2	0	2	0	E
44	2	2	0	0	0	0	0	0	0	UN	1	2	2	0	2	1	2	2	2	0	0	NA	0	0	2	0	0	NA	0	2	2	E
53	2	2	2	2	2	UN	2	2	0	2	1	2	2	2	2	1	2	2	2	0	0	NA	2	0	0	0	0	NA	0	2	0	E
64	2	2	2	2	2	UN	0	2	0	2	2	2	2	2	2	2	2	2	2	0	0	NA	2	0	0	0	0	NA	0	2	0	E
81	2	2	2	2	0	0	0	2	0	2	0	0	0	NA	2	1	2	2	2	0	0	NA	2	0	0	0	0	NA	0	2	2	E
82	1	2	2	1	0	UN	2	2	2	0	1	2	2	2	2	2	2	2	2	2	0	NA	2	0	1	0	0	NA	0	2	2	E
89	1	0	2	0	0	UN	0	2	0	2	0	2	0	NA	0	0	2	2	2	0	0	NA	2	0	0	0	0	NA	0	0	2	E
99	2	2	2	2	0	0	2	2	2	2	2	0	2	2	2	2	2	2	2	2	0	NA	2	0	2	0	0	NA	0	2	0	E
107	2	2	2	2	0	0	2	2	2	2	1	2	0	NA	2	1	2	2	2	2	0	NA	0	0	0	0	0	NA	0	2	0	E
108	2	2	2	2	0	0	2	2	2	2	2	0	0	NA	2	2	2	2	2	0	2	0	2	0	0	0	0	NA	0	2	0	E
109	1	1	2	1	0	2	0	0	0	2	1	2	0	NA	2	1	2	2	2	0	0	NA	2	0	0	0	0	NA	0	0	2	E
114	2	2	2	2	2	2	2	2	2	2	2	2	0	NA	2	2	2	0	0	0	0	NA	2	0	0	2	0	NA	0	0	0	E
115	1	2	NA	0	2	2	2	2	0	2	UN	2	2	2	0	0	2	0	0	0	0	NA	0	2	0	0	2	0	0	0	0	E;CJ
74	2	2	2	0	0	UN	0	2	0	2	2	0	2	2	2	0	0	2	0	2	0	NA	2	0	0	0	0	NA	0	2	0	E;D
7	2	0	2	1	0	0	2	2	0	2	2	0	2	2	0	0	2	2	0	2	UN	UN	2	0	0	0	0	NA	0	0	2	E;ID;SW
15	1	2	2	1	0	UN	2	2	0	2	1	0	2	2	2	2	2	2	0	2	2	2	2	0	0	0	0	NA	0	0	0	E;SW
20	1	1	2	2	2	2	2	2	0	2	1	2	0	NA	0	0	2	2	2	0	2	2	0	0	0	0	0	NA	0	0	0	E;SW
24	2	1	2	0	0	UN	2	2	0	2	1	2	0	2	2	0	2	0	0	0	0	NA	2	0	0	0	0	NA	0	2	0	E;SW
2	1	0	2	2	0	0	2	2	0	2	0	0	0	NA	0	2	0	2	2	0	2	2	0	0	0	0	0	NA	0	0	2	ID
3	1	0	2	0	0	0	2	2	0	2	2	0	2	2	0	0	2	0	0	0	2	2	2	0	0	0	0	NA	0	2	0	ID
6	1	0	1	0	0	0	2	2	0	2	1	0	0	NA	0	0	2	0	0	0	2	2	2	0	0	0	0	NA	0	0	0	ID
13	2	2	2	2	0	0	2	2	0	2	2	0	2	2	0	1	0	2	0	0	0	NA	2	2	2	0	0	NA	0	0	2	ID
25	2	2	2	1	0	0	2	2	0	2	1	0	0	NA	0	0	2	2	2	0	UN	UN	0	0	0	0	0	NA	0	2	2	ID
34	2	2	2	2	0	0	2	2	1	2	2	0	2	2	2	2	2	0	0	0	2	2	0	0	0	0	2	2	0	2	2	ID
40	2	2	2	2	0	0	2	2	1	2	2	0	2	2	2	1	2	0	0	0	2	2	2	0	0	0	0	NA	0	2	0	ID
41	1	1	1	0	2	UN	2	2	0	2	2	2	0	NA	0	0	2	0	0	0	0	NA	0	0	0	0	0	NA	0	0	0	ID

**PRISMA-S**	1,2	1	3		3	3				5	13	12	9	9	8	8	5	4	4	4	5	5	6	6	9	10	11	11	14	15	16	
**PRISMA**	6		6		6	6				6	6				7	7	6															
**MECCIR**	R34										R35		R36		R38	R39																
Study ID	db_list	db_platform	gray_list	gray_url	registries	trials	govt	ngo	date_range	backward	date_searches	update_search	limit_date	limit_just	strategy_all	gray_search	reviews	handsearch	hand_journ	hand_conf	forward	forward_method	0	experts_listserv	limit_lang	hedge	adapt	adapt_cite	peer_review	num_records	deduplicate	Coordinating Groups
47	1	0	2	0	0	0	2	2	0	2	0	0	2	2	2	1	0	0	0	0	0	NA	2	0	1	0	0	NA	0	0	0	ID
48	1	2	2	0	0	0	2	2	0	2	0	0	2	2	UN	0	0	0	0	0	2	2	0	0	0	0	0	NA	0	2	2	ID
54	2	1	1	2	2	2	2	2	2	2	2	0	0	NA	0	0	2	0	0	0	2	2	2	0	0	0	0	NA	0	0	0	ID
65	1	2	2	2	0	0	2	2	0	2	2	0	2	2	2	2	0	0	0	0	0	NA	2	0	2	0	2	2	0	2	2	ID
66	1	0	2	0	2	2	2	2	0	2	0	0	2	2	0	2	2	0	0	0	0	NA	2	0	0	0	0	NA	0	0	0	ID
75	2	2	2	2	0	0	2	2	1	2	2	0	2	2	0	2	2	0	0	0	2	2	2	0	0	0	0	NA	0	0	2	ID
76	2	2	2	0	0	0	2	2	0	2	2	0	2	2	2	2	0	0	0	0	2	2	2	0	1	0	0	NA	0	0	0	ID
77	2	2	2	2	0	0	2	2	0	2	1	0	2	2	0	2	2	0	0	0	2	2	2	0	0	0	0	NA	0	0	2	ID
83	1	2	2	1	2	0	2	2	0	2	1	2	2	2	0	0	2	0	0	0	0	NA	2	0	0	0	0	NA	0	2	0	ID
84	2	2	2	2	0	0	2	2	0	2	2	0	2	2	0	2	2	2	2	0	2	2	2	UN	0	0	0	NA	0	UN	2	ID
104	2	1	2	1	2	2	2	2	1	2	2	0	0	NA	0	2	2	2	2	0	0	NA	2	0	0	2	2	2	0	0	2	ID
116	1	0	2	2	0	UN	2	2	0	2	0	0	UN	NA	0	0	2	0	0	0	2	2	2	0	UN	0	0	NA	0	0	0	ID
118	2	2	2	2	2	2	2	2	2	2	2	0	0	NA	0	0	2	0	0	0	0	NA	2	0	0	0	0	NA	0	0	0	ID
43	2	2	2	0	2	2	2	2	1	2	2	0	0	NA	2	0	2	0	0	0	0	NA	2	0	0	0	2	2	0	0	0	ID;N
50	1	1	NA	2	2	2	0	0	0	2	2	0	0	0	0	0	0	0	0	0	2	2	0	0	0	0	0	NA	0	0	0	ID;N
61	2	0	2	0	2	2	2	2	0	2	2	2	0	UN	2	0	2	0	0	0	2	2	0	0	0	0	0	NA	0	0	0	ID;N
67	2	2	2	2	UN	2	2	2	0	2	2	0	0	NA	0	1	2	0	0	0	0	NA	2	0	0	0	0	NA	0	0	0	ID;N
68	2	2	2	1	2	2	2	2	0	2	2	0	0	NA	0	0	2	0	0	0	UN	UN	2	0	0	0	0	NA	0	2	0	ID;N
21	2	1	2	2	0	2	2	2	1	2	1	0	0	NA	2	0	2	0	0	0	0	NA	2	0	0	0	2	2	0	0	0	KTI
35	2	2	2	0	0	0	0	2	0	2	1	0	0	NA	2	2	2	0	0	0	2	2	2	0	0	0	0	NA	0	2	0	M
1	1	2	2	NA	2	2	0	0	1	2	2	0	2	2	2	0	0	0	0	0	0	NA	2	0	0	2	0	NA	0	0	0	SW
14	2	2	2	0	2	2	0	0	2	2	2	2	0	2	2	0	2	0	0	0	0	NA	2	0	0	2	0	NA	0	0	0	SW
16	2	2	2	2	0	2	2	2	0	2	2	2	0	NA	2	1	2	2	2	0	0	NA	2	0	0	0	0	NA	0	0	0	SW
26	2	2	2	2	0	0	2	2	0	2	2	2	0	NA	2	1	2	2	2	0	0	NA	0	0	0	0	0	NA	0	2	0	SW
27	2	2	2	NA	0	2	0	0	0	2	2	2	2	2	2	0	0	0	0	0	0	NA	2	0	0	0	0	NA	0	2	0	SW
45	2	2	2	1	0	0	2	2	2	2	2	2	2	2	2	2	2	0	0	0	0	NA	2	0	0	0	0	NA	0	2	2	SW
46	2	2	2	2	2	0	2	2	2	2	1	2	0	NA	0	0	2	2	2	2	2	2	0	2	0	0	0	NA	0	0	0	SW
58	2	2	2	0	2	2	2	2	0	2	2	2	0	NA	2	0	2	2	2	0	0	NA	2	0	0	0	0	NA	0	2	0	SW
60	2	2	2	2	0	0	2	2	0	2	2	0	0	NA	2	1	2	2	2	0	0	NA	2	0	0	0	0	NA	0	2	0	SW
71	2	2	0	NA	0	2	0	0	1	2	2	0	0	0	2	0	0	0	0	0	0	NA	0	0	0	0	0	NA	0	0	0	SW
78	2	0	2	1	0	2	2	0	2	0	2	2	2	2	2	2	2	0	0	0	0	NA	2	0	0	2	2	2	0	0	0	SW
85	2	2	2	2	2	0	2	2	2	2	1	2	0	NA	2	2	2	2	2	0	2	2	2	0	0	0	0	NA	0	0	0	SW
86	2	2	2	2	0	0	0	0	0	2	2	0	0	NA	2	2	2	2	2	0	0	NA	0	0	0	0	0	NA	0	2	2	SW

Study ID	db_list	db_platform	gray_list	gray_url	registries	trials	govt	ngo	date_range	backward	date_searches	update_search	limit_date	limit_just	strategy_all	gray_search	reviews	handsearch	hand_journ	hand_conf	forward	forward_method	0	experts_listserv	limit_lang	hedge	adapt	adapt_cite	peer_review	num_records	deduplicate	Coordinating Groups
**PRISMA-S**	1,2	1	3		3	3				5	13	12	9	9	8	8	5	4	4	4	5	5	6	6	9	10	11	11	14	15	16	
**PRISMA**	6		6		6	6				6	6				7	7	6															
**MECCIR**	R34										R35		R36		R38	R39																
88	1	1	2	0	0	2	2	2	1	0	2	0	0	0	2	2	2	0	0	0	0	NA	0	0	0	2	0	NA	0	0	0	SW
90	2	2	2	2	0	UN	2	2	0	2	2	2	0	NA	2	2	2	2	2	0	0	NA	0	0	0	0	0	NA	0	2	0	SW
92	1	1	2	1	0	0	2	2	0	0	1	0	2	2	2	0	2	0	0	0	0	NA	2	0	0	0	0	NA	0	2	0	SW
98	2	0	1	1	2	0	UN	2	0	2	2	2	2	2	2	1	0	0	0	0	0	0	2	0	2	0	0	NA	0	2	0	SW
100	2	2	2	2	2	2	2	2	0	2	2	2	2	2	2	2	2	2	2	2	2	2	2	0	0	0	0	NA	0	0	2	SW
101	2	2	2	2	0	0	0	2	0	2	2	0	0	NA	2	2	2	2	2	0	UN	UN	2	0	0	0	0	NA	0	2	2	SW
105	2	2	1	0	2	UN	2	2	0	2	1	2	0	NA	0	2	2	2	2	0	0	NA	2	0	0	0	0	NA	0	0	0	SW
106	2	2	2	2	2	UN	2	2	0	2	2	0	2	2	2	2	2	0	0	0	0	NA	2	0	0	0	0	NA	0	0	0	SW
110	2	2	2	1	2	2	2	2	2	2	2	2	0	NA	2	2	2	0	0	0	0	NA	2	0	0	0	0	NA	0	2	0	SW
119	2	2	2	1	0	2	UN	UN	1	2	2	0	2	0	2	1	2	0	0	0	2	2	2	0	0	0	0	NA	0	2	0	SW
121	2	2	2	2	0	0	2	2	1	2	1	0	2	0	0	2	2	2	2	UN	0	NA		0	0	0	0	NA	0	0	0	SW
17	2	0	1	1	0	2	2	2	0	2	0	0	2	2	0	0	2	2	2	0	0	NA	2	0	0	0	0	NA	0	0	0	SW

Figure S2. Heatmap summary of adherence to reporting standards

Colours (indicate degree of agreement with variables): blue (complete - 2), yellow (partial - 1), red (none - 0), grey (unclear), white (not applicable - NA)

Coordinating Groups: A (Aging): BM (Business and Management); CJ (Criminal Justice); D (Disability); E (Education); ID (International Development); N (Nutrition); KTI (Knowledge Translation and Implementation); M (Methods); SW (Social Welfare)

Standards: PRISMA 2020 (Preferred Reporting Items for Systematic Reviews and Meta-Analyses); PRISMA-S (PRISMA extension for searching); MECCIR (Methodological Expectations for Campbell Collaboration Intervention Reviews, 2019)

Study Variables: See Supplementary File 1: Data extraction form and codebook
